# Midkine signaling maintains the self-renewal and tumorigenic capacity of glioma initiating cells

**DOI:** 10.7150/thno.41450

**Published:** 2020-04-06

**Authors:** Israel López-Valero, David Dávila, José González-Martínez, Nélida Salvador-Tormo, Mar Lorente, Cristina Saiz-Ladera, Sofía Torres, Estibaliz Gabicagogeascoa, Sonia Hernández-Tiedra, Elena García-Taboada, Marina Mendiburu-Eliçabe, Fátima Rodríguez-Fornés, Rebeca Sánchez-Domínguez, José Carlos Segovia, Pilar Sánchez-Gómez, Ander Matheu, Juan M. Sepúlveda, Guillermo Velasco

**Affiliations:** 1 Department of Biochemistry and Molecular Biology, School of Biology, Complutense University, Madrid, Spain; 2 Instituto de Investigaciones Sanitarias San Carlos (IdISSC), 28040 Madrid, Spain; 3 Instituto Universitario de Investigación Neuroquímica, Complutense University, 28040 Madrid, Spain; 4 Centro de Investigación Biomédica en Red Sobre Enfermedades Neurodegenerativas.; 5 Division of Hematopoietic Innovative Therapies, Centro de Investigaciones Energéticas, Medioambientales y Tecnológicas, Madrid, Spain.; 6 Advanced Therapies Unit, Instituto de Investigación Sanitaria Fundación Jiménez Díaz, Madrid, Spain.; 7 Centro de Investigación Biomédica en Red de Enfermedades Raras, Madrid, Spain.; 8 Neuro-oncology Unit, Instituto de Salud Carlos III, Majadahonda, Madrid, Spain; 9 Cellular Oncology group, Biodonostia Health Research Institute, Spain; 10 CIBER de Fragilidad y Envejecimiento Saludable (CIBERfes), Madrid, Spain; 11 IKERBASQUE, Basque Foundation, Bilbao, Spain.; 12 Neuro-oncology Unit, Hospital Universitario 12 de Octubre, Madrid, Spain

**Keywords:** glioblastoma, Midkine, ALK receptor tyrosine kinase, autophagy, SOX, combinational therapies

## Abstract

Glioblastoma (GBM) is one of the most aggressive forms of cancer. It has been proposed that the presence within these tumors of a population of cells with stem-like features termed Glioma Initiating Cells (GICs) is responsible for the relapses that take place in the patients with this disease. Targeting this cell population is therefore an issue of great therapeutic interest in neuro-oncology. We had previously found that the neurotrophic factor MIDKINE (MDK) promotes resistance to glioma cell death. The main objective of this work is therefore investigating the role of MDK in the regulation of GICs.

**Methods**: Assays of gene and protein expression, self-renewal capacity, autophagy and apoptosis in cultures of GICs derived from GBM samples subjected to different treatments. Analysis of the growth of GICs-derived xenografts generated in mice upon blockade of the MDK and its receptor the ALK receptor tyrosine kinase (ALK) upon exposure to different treatments.

**Results**: Genetic or pharmacological inhibition of MDK or ALK decreases the self-renewal and tumorigenic capacity of GICs via the autophagic degradation of the transcription factor SOX9. Blockade of the MDK/ALK axis in combination with temozolomide depletes the population of GICs *in vitro* and has a potent anticancer activity in xenografts derived from GICs.

**Conclusions**: The MDK/ALK axis regulates the self-renewal capacity of GICs by controlling the autophagic degradation of the transcription factor SOX9. Inhibition of the MDK/ALK axis may be a therapeutic strategy to target GICs in GBM patients.

## Introduction

Grade IV astrocytoma, or glioblastoma (GBM) is one of the most aggressive forms of cancer. GBM patients typically exhibit a median survival after diagnosis of 12-15 months 1, 2. These tumors frequently exhibit molecular alterations in several oncogenes and tumor suppressor genes affecting some of the most relevant signaling pathways involved in the control of tumorigenesis, including through tyrosine kinase receptors (RTKs). Enhanced RTK signaling is thought to contribute to the high invasiveness and resistance to most anticancer therapies exhibited by GBMs 1, 2. The standard treatment for these tumors is based on surgical resection, which frequently is not complete due to the proximity of the tumor mass to vital brain regions, followed by radiotherapy and concomitant chemotherapy with the alkylating agent temozolomide (TMZ) 1-3. Unfortunately, almost all patients with GBM relapse after a few months. The identification within these tumors of a restricted cell population with characteristics of stem cells [termed Glioma stem-like cells or Glioma Initiating Cells (GICs)] exhibiting a high resistance to present anti-glioma therapies led to postulate the currently-accepted theory that GICs are directly involved in GBM relapse. Therefore one of the strategies to improve the survival of patients with this disease should be based on elimination of GICs 2, 4-7.

The neurotrophic and developmental factor MIDKINE (MDK) belongs to the PLEIOTROPHIN/ MIDKINE family and has been shown to participate in the regulation of many different physiological functions 8-10. Specifically, MDK has been implicated in the regulation of neural stem cells and embryonic CNS development 11. MDK effects have been proposed to rely on the stimulation of several different receptors 10 although a significant number of the functions of the protein has been attributed to its ability to stimulate the ALK receptor tyrosine kinase (ALK) 10, 12. In a cancer context, MDK has been proposed to promote resistance to anticancer treatments via ALK stimulation 13. In addition, MDK levels have been shown to be increased in different cancers 14-18. Specifically, our group and others previously demonstrated that enhanced MDK levels correlated with worse prognosis in GBM patients 13, 19, thereby suggesting that this growth factor may play a role in glioma generation and progression.

In this work we investigated whether the MDK/ALK axis is involved in the regulation of GICs biology. Our findings demonstrate that MDK plays a key role in the maintenance of the stem-like and tumorigenic properties of GICs via ALK stimulation. We also found that inhibition of the MDK/ALK axis may be used as a therapeutic strategy to target the population of GICs as treatment for GBM.

## Materials and Methods

### Reagents

Mouse monoclonal anti-MDK antibody (N-terminal region, MDK Ab.) and purified recombinant MDK were kindly provided by LYRAMID (Sydney, Australia). Both reagents were resuspended in PBS. Doxycycline (Dox) and puromycin were purchased from Sigma (St. Louis, MO, USA) and resuspended in DMEM and PBS, respectively. NVP-TAE 684 (TAE) was kindly provided by Sergey A. Lakatosh at the Gause Institute of New Antibiotics (Moscow, Russia) or purchased from MedChem Express (Sollentuna, Sweeden). Crizotinib and lorlatinib were kindly donated by Pfizer (New York, NY, USA). Pure THC and CBD were obtained from THC Pharm Company (Frankfurt, Germany). TMZ and lactacystin were purchased from Calbiochem-Merck (Darmstadt, Germany). E64d and pepstatin A (PA) were purchased from Enzo Life Science (Farmingdale, NY, USA).

### Cell culture

Glioblastoma patient-derived cultures enriched in cells with stem-like properties (that in this work we named cultures of Glioma Initiating Cells, GICs) were obtained from human GBM tumor samples from the Spanish National Cancer Center (CNIO, Madrid, Spain) biobank (GH2 and GH11-GICs), from Hospital 12 de Octubre (Madrid, Spain) (12O12-GICs) and from Hospital Clinico San Carlos (Madrid, Spain) (HCO1-GICs). All procedures involving samples of human origin were performed with the approval of the corresponding ethical committees from each institution as well as of the ethical committee of Complutense University (Madrid, Spain). Briefly, GICs cultures were obtained by using the following procedure: tumor samples were mechanically and enzymatically dissociated with 0.12 mg/ml of collagenase type Ia from *Clostridium histolyticum* (#C9722, Sigma) for 2 h at 37 ºC and filtered using a 100 μm nylon filter (Millipore, Burlington, MA, USA). Cells were then plated and maintained as non-adherent neurospheres for at least 3 consecutive passages (with the aim of enriching the cultures in cells with stem-like properties) in a DMEM:Ham's F-12 media (Lonza, Basel, Switzerland) supplemented with 1% penicillin-streptomycin (Lonza), 5 mM HEPES buffer (Lonza), 2 mM ultraglutamine (Lonza), 20 ng/ml EGF and FGFb (Gibco, Carlsbad, USA), 2 μg/ml heparin sodium salt (Sigma), 1% B27 (Invitrogen, Carlsbad, USA) and 1 µg/ml leukemia inhibitory factor (LIF, Millipore). Enrichment in GICs was analyzed by testing the expression of a panel of stem cell markers in these cultures. To induce differentiation of GICs, supplements were removed and cells were cultured in DMEM containing 10% FBS and 1% penicillin-streptomycin.

HEK293T and the human glioma U87MG (ATCC® HTB-14™) cell lines were purchased from ATCC (Manassas, Virginia, USA) and cultured in DMEM containing 10% FBS and 1% penicillin-streptomycin. U87-neurospheres cultures (U87-GICs) were generated by incubation in DMEM:Ham's F-12-supplemented media as described above and maintained as non-adherent cultures for at least 3 consecutive passages. All cell cultures were incubated at 37 ºC, 5% CO_2_. Experiments were performed using GICs cultures between passages 3 and 20.

Unless otherwise indicated, drugs were prepared in DMSO for *in vitro* experiments. Control incubations contained the same amount of DMSO and no significant effect was observed in any of the parameters determined throughout this study at the final concentration used (< 0.5%, v/v).

### Analysis of tumor-sphere growth capacity of GICs cultures

Cultures of GICs were plated at a density of 10^4^ cells/ml (passage 0, P0) and incubated with the different treatments for 5 days. The spheres formed were then dissociated, counted (passage 1, P1) and equal number of cells re-plated and incubated again with the corresponding treatments for 5 additional days. This procedure was repeated for two consecutive passages (passage 2, P2). Data from these experiments are expressed as the total number of cells counted upon disaggregation of spheroid cultures in each passage and are represented as the mean fold-change from the number of cells plated at P0.

### Limiting Dilution Assays (LDA)

Limiting dilution assays were performed as previously described 20. Briefly, cultures of GICs were plated at density of 10^4^ cells/ml and incubated with the different treatments for 5 days. Spheres formed were dissociated and plated in fresh medium in the absence of the drug in 96-well plates at different densities (200, 100, 50, 20 and 10 cells per well, respectively). One week later, tumorsphere formation was evaluated: wells in which there was at least one neurosphere were considered positive. Data in the corresponding representations indicates the fraction of cells with ability to generate new spheres cultures. Graphs were obtained using the ELDA software application 20 that adjusts the data obtained in each experimental condition to the limiting dilution model. In these graphs the slopes of the depicted solid lines correspond to the fraction of cells with ability to generate new spheres cultures. A lower slope value indicates a lower fraction of cells with capacity to generate new spheres. Dotted lines represent the 95% confidence interval.

### Human MIDKINE ELISA detection

Soluble MDK levels were determined by ELISA Kit for human MDK detection (LYRAMID) according to manufacturer's instructions.

### Quantitative real-time PCR

RNA was isolated by using the Trizol reagent (Invitrogen) following manufacturer's instructions and including a DNAse digestion step with the RNAse-free DNAse kit (Qiagen, Maryland, USA). cDNA was obtained from 1 µg of RNA using the Transcriptor Reverse Transcriptase (Roche, Basel, Switzerland). Real-time quantitative PCR assays were performed using the FastStart Master Mix (Roche) and probes were obtained from the Universal Probe Library Set (Roche). Primer sequences and Roche's probes corresponding to each pair of primers can be found in Supplementary Table 1 (Methods Information). Amplifications were run in a 7900 HT-Fast Real- Time PCR System (Applied Biosystems, Carlsbad, CA, USA). Each value was normalized using 18S, TBP or GAPDH RNA levels as reference. Gene expression was quantified by the delta-delta Ct method.

### Light field microscopy

Images were obtained in an Eclipse TE300 microscope (Nikon, Minato, Tokyo, Japan) coupled to a digital camera DS-U2 (Nikon).

### Immunofluorescence staining and confocal microscopy

Neurospheres were plated onto glass cover slips pre-treated with Matrigel (BD Bioscience, NY, USA, dilution 1:100 in DMEM medium), fixed with 4% paraformaldehyde (Sigma) for 10 min, permeabilized and blocked with 10% goat serum PBS containing 0.25% Triton X-100 (Sigma) for 1 hour and incubated with the corresponding primary antibodies Supplementary Table 2 (Methods Information)] in 5% goat serum overnight at 4 ºC. Cover slips were washed with PBS and incubated with the corresponding anti-rabbit or anti-mouse Alexa-488 or Alexa-594-conjugated secondary antibodies (Life Technologies, Carlsbad, CA, USA) at a 1:1000 (v/v) dilution at room temperature for 1 hour and nuclei were stained with DAPI (Roche) for 10 min. Finally, cover slips were mounted with mowiol (Calbiochem- Merck) and images were obtained in a Leica TCS SP2 confocal microscope (Leica, Germany).

Samples from tumor xenografts were dissected, frozen in Tissue-Tek OCT compound (Sakura, CA, USA) and cryostat sectioned (8 μm) using a Leica CM 1950 cryostat (Wetzlar, Germany). Sections were fixed in acetone (Panreac, Darmstadt, Germany) for 10 min at room temperature, permeabilized and blocked as previously described and incubated with the corresponding primary antibodies (Supplementary Table 2 (Methods Information)). Images were obtained in an Axiovert 135 microscope (Carl Zeiss, Thornwood, NY, USA).

### TUNEL assay

TUNEL was performed as previously described [21 using the *in situ* Cell Death Detection Kit (Sigma).

### Quantification of LC3 vesicles

The quantification of LC3 vesicles was performed using the ImageJ JavaScript AUTOCONTER as previously described 22. The ratio between the sum of the LC3 vesicle areas (ΣAves) and the total cell area (Acell) was calculated per each cell. At least 11 cells per treatment from 3 independent experiments were analyzed.

### Western blot

Western blot analysis was performed following standard methods. Briefly, cells were lysed in a buffer containing 50 mM Tris-HCl, 0.1% Triton X-100, 50 mM NaF, 10 mM sodium glicerophosphate, 5 mM sodium pyrophosphate, 1 mM sodium orthovanadate, 1µg/ml leupeptine, 1 mM EDTA and EGTA, 200 µM β-mercaptoetanol and 1 µM microcystin. Tumor samples were lysed on RIPA buffer supplemented with 1 mM sodium orthovanadate, 0.1 mM phenylmethylsulfonyl fluoride (PMSF), 2 µg/µl aprotinin and 2 µg/µl leupeptin. Lysates were centrifuged at 12,000 rpm for 15 min. Protein concentration was determined by Bradford assay. Proteins were electrophoretically separated in SDS-polyacrylamide gels (Biorad, Hercules, CA, USA), transferred onto PVDF membranes (Biorad), blocked with a 5% skimmed milk solution or 5% bovine serum albumin (BSA, Sigma) in TBS-T [10 mM Tris-HCl pH 7.5, 100 mM NaCl, and 0.1% Tween-20 (Panreac)] for 1 h at room temperature and incubated overnight at 4 ºC with the corresponding primary antibody (a list of the primary antibodies used in the Western blot analyses included in this study can be found in Supplementary Table 3 (Methods Information)). The day after, membranes were washed with TBS-T and incubated for 1 h at room temperature with their corresponding secondary antibody [HRP-conjugated anti-rabbit or anti-mouse (GE Healthcare Dharmacon, Chicago, USA)]. Immunoreactivity was detected using the enhanced chemiluminescence (ECL) system (Biorad). Densitometric quantification was performed with ImageJ software.

### Immunoprecipitation

Cells were lysed using a HEPES lysis buffer containing 40 mM HEPES pH 7.5, 120 mM NaCl, 1mM EDTA, 10 mM sodium pyrophosphate, 10 mM sodium glycerophosphate, 50 mM sodium fluoride, 0.5 mM sodium orthovanadate, 0.3% CHAPS, and supplemented with 1 mM benzamidine and 0.1 mM PMSF freshly added. Cell lysates (1-2 mg) were precleared by incubation with 10 μl of protein G-sepharose (GE Healthcare Dharmacon) at 4 ºC for 30 min. The lysate extracts were then incubated overnight at 4 ºC with 5-10 μl of protein G-sepharose covalently coupled to 5-10 μg of the primary anti-LC3 (ref. L8918, Sigma) or anti-SOX9 (ref. ab76997, Abcam) antibodies or an unspecific IgG (negative control). Immunoprecipitates were washed 4 times with HEPES lysis buffer followed by 2 washes with HEPES kinase buffer (25 mM HEPES pH 7.5 and 50 mM KCl), resuspended in 30 μl of sample buffer (without 2-mercaptoethanol) and filtered through a 0.22 μm-pore size Spin-X filter (Sigma). Then 2-mercaptoethanol was added to a concentration of 1% (v/v). Samples were subjected to electrophoresis and immunoblot analysis following standard procedures.

### Lentiviral particles generation and transduction

For lentiviral particle generation (shATG5), HEK293A were plated in 60 cm^2^ dishes and transfected with a human TRIPZ ATG5 lentiviral doxycycline-inducible shRNA plasmid (clone ID V2THS_249282, Thermo Scientific, Waltham, MA, USA) using the standard calcium-phosphate transfection protocol 23. 48 h later, supernatant was collected, filtered using a 0.45 µm diameter low protein binding filter (Millipore) and mixed up with Lenti-X™ Concentrator (Clontech, Fremon, CA, USA) at a 1:3 (v/v) ratio overnight at 4 ºC. Lentiviral particles were precipitated, resuspended in DMEM and stored at -80 ºC. Titer of lentiviral particle stocks was determined by plating HEK293T cells in 24-well plates transfected with serial dilutions of the viral stock and subsequent counting of the number of Turbo GFP or RFP positive cells for each well.

Lentiviral vectors encoding target-specific doxycycline-inducible shRNA constructs targeting MDK (Thermo Scientific; SMART choice inducible lentiviral shRNA) or ALK (GE Healthcare Dharmacon; SMARTvector human inducible shRNA) were used. The three sequences provided for targeting MDK were GCTCGTTAGCTTTAATCAA (shMDK23), TTGGAGCCGACTGCAAGTA (shMDK31) and GGGATTCTGGGAAGCTTGA (shMDK39). After analysis of the silencing efficacy of each of these sequences, shMDK39 was selected. The two sequences provided for targeting ALK were TAGTTGGGGTCATAGATGT (shALK03) and CCTGTGAGCTGGAGTATTC (shALK06). After analysis of the silencing efficacy of each of these sequences, shALK03 was selected.

To stably knock-down the expression of ATG5 in GICs-cultures we used a SMART-pool of concentrated transduction-ready human lentiviral particles containing 3 target-specific inducible shRNA constructs [mature antisense: AAGTTTCTGAGATTGTATG, TTCGTTAAGGAAAGATGGG and ATCTCACTAATGTCTTCTT)] (ref. RHS11852-EG9474, GE Healthcare Dharmacon) or a SMART-pool of concentrated transduction-ready human lentiviral particles containing 3 target-specific constitutive shRNA constructs (sc-41445-V, Santa Cruz Biotechnology, Dallas, Texas, USA). To stably knock-down ATG7 we used a SMART-pool of concentrated transduction- ready human lentiviral particles containing 3 target- specific constitutive shRNA constructs (sc-41447-V, Santa Cruz Biotechnology). A doxycycline-inducible (GE Healthcare Dharmacon) or a constitutive (Santa Cruz Biotechnology) scrambled shRNA construct were used as controls.

Briefly, GICs cultures were plated at a density of 10^4^ cells/ml in a supplemented GIC media with hexadimethrine bromide (Sigma) at a final concentration of 2.5 mg/ml. Cells were subsequently infected with control- or target-specific shRNA lentiviral particles. Two days later, the medium was removed and replaced by complete medium without hexadimethrine bromide. Finally, to select the clones stably expressing the shRNAs, the cells were incubated with puromycin (Gibco) at a concentration of 1 to 5 µg/ml. To induce shRNA expression, doxycycline (Dox) at a final concentration of 1 µg/ml was added. All experiments with cells transduced with doxycycline-inducible shRNAs were performed in cell cultures that had been exposed to doxycycline for at least two passages to ensure the appropriate expression of the corresponding shRNAs.

### Nucleofection with plasmids

For SOX9 knock-down or overexpression, GICs-cultures were nucleofected with a plasmid expressing a SOX9-selective shRNA [(pLKO shSOX9 (#40644, Addgene, Cambridge, MA, USA)] or a plasmid encoding a murine form of SOX9 [(pWPXL SOX9 (#36979, Addgene)] respectively. A pLKO plasmid expressing a scramble shRNA (#8453, Addgene) or a pWPXL GFP (#12257, Addgene) were used as controls (control plasmids, CP). Briefly, 5 x 10^5^ GICs were plated, centrifuged at 1100 rpm for 5 min, resuspended with 50 µl of nucleofector kit (Lonza) and electroporated with 3 µg of the corresponding plasmid using the Nucleofector^TM^ 2b Device (Lonza). All experiments with plasmid-nucleofected cells were performed at least 4 days after electroporation to ensure the appropriate shRNA or protein expression was taking place.

### Flow cytometry

(0.2-1) x 10^6^ GICs for each experimental condition were fixed with 4% paraformaldehyde (Sigma) for 10 min, permeabilized with Perm/Wash buffer 1x (BD Bioscience) for 20 min and subsequently incubated with the Nestin-PE (ref.656805, clone 10C2, Biolegend, San Diego, CA, USA) antibody for 30 min at 22 ºC in darkness. Finally, samples were washed with the Perm/Wash buffer 1x and analyzed by flow cytometry (LSR Fortessa, BD Bioscience/Becton Dickonson and Company, New Jersey, USA) using the FlowJow software v 7.6.5 (Tree star, Ashland, USA).

### Generation of tumor xenografts and drug treatment

For subcutaneous xenografts, 2 x 10^6^ 12O12-GICs resuspended 1:1 (v/v) in 100 μl of supplemented GICs-medium and matrigel (BD Bioscience) were subcutaneously injected in the right flank of 5-week-old female nude mice (Harlan Laboratories, Indiana, USA). Tumors were daily measured with an external caliper, and volume was calculated as (4π/3) x (width/2)^2^ x (length/2). When tumors had reached an average size of 200 mm^3^, animals were randomly assigned to different groups and treatments with the corresponding drugs commenced.

Crizotinib and lorlatinib were diluted in PBS or water with a pH range of 2.75-3.25 respectively and orally administered by using an oral gavage. TMZ was diluted in PBS supplemented with 5 mg/ml BSA and intraperitoneally (I.P.) administered. The corresponding diluent was used as vehicle for each treatment. Once the treatments were completed, animals were sacrificed, and tumors excised for further analyses.

For the generation of intracranial xenografts, 12O12-GICs with less than 3 passages were infected with lentiviral particles expressing a shC (control), shMDK or shALK and subsequently selected using puromycin (1 µg/ml). One week before injection, shRNA expression was induced by treatment with doxycycline (1 µg/ml). 0.75 x 10^5^ infected 12O12-GICs resuspended in 4 μl of supplemented GIC-medium were stereotactically injected into the right cerebral hemisphere of nude mice (coordinates A-P: +1 mm; M-L: -2 mm; D-V: -3 mm relative to bregma) by using a 10 µl Hamilton syringe 701RN (Hamilton, Nevada, USA). Animals were previously anesthetized with isoflurane and subsequently treated with a mixture of buprenorphine (0.1 mg/kg) and meloxicam (1 mg/kg). To ensure that shRNA expression was maintained along the *in vivo* experiment, animals injected with 12O12 GICs shMDK/shALK (+Dox) were fed with a doxycycline-enriched diet (Sodispan, Madrid, Spain). The monitoring of tumor growth by magnetic resonance imaging (MRI) was performed at the Nuclear Magnetic Resonance Centre of Complutense University (Madrid, Spain) using a BIOSPEC BMT 47/40 (Bruker, Ettlingen, Germany). Tumor volume was calculated using the ImageJ software from T1-weighted images.

### Patient dataset and bioinformatics analysis

Kaplan-Meier survival analysis plots were obtained from “GlioVis” 24, a website application for data visualization and analysis to explore gene expression datasets from studies involving patients with brain tumors. This tool expresses the 'survival' package in R. The following datasets were evaluated to correlate MDK expression and patient survival: Rembrandt study 25 (microarray data, containing information from 225 low-grade glioma and 219 GBM samples, respectively); Gravendeel study 26 (microarray data, containing information from 117 low-grade glioma and 159 GBM samples, respectively); Lee Y. study 27 (microarray data type, containing information from 191 GBM samples).

Additional survival analysis in correlation with MDK expression were obtained using the “The Pathology Atlas” section of the “Human Protein Atlas” 28. More than 100 million of Kaplan-Meier plots were analyzed by the “Human Protein Atlas” portal to correlate gene expression levels and survival time. Median expression levels were used as a cutoff. Best separation cutoffs are presented for each gene in each cancer type.

### Statistical analysis

Unpaired Student's t test or U Mann-Whitney test were used when two independent groups were analyzed. For multiple comparison, analysis of variance (ANOVA) test was performed (one-way or two-way ANOVA) with a Tukey's post hoc test. χ²-test was used for LDA experiments. The survival of nude mice was analyzed by Kaplan-Meier curves and differences were compared by log-rank test analysis. Data are expressed as mean ± standard error of the mean (SEM). P values of < 0.05 were considered statistically significant. Analyses were performed using the GraphPad Prism 6.0 software.

### Study approval

All procedures involving samples of human origin were performed with the approval of the corresponding ethical committees from each institution as well as of the ethical committee of Complutense University/Hospital Clínico San Carlos. Samples from GBM patients used in this study were originally obtained upon signature of the corresponding written informed consent (which had been previously approved by the Institutional Ethical Committee). Samples were incorporated in the corresponding institutional biobanks. All procedures involving animals were performed with the approval of the corresponding ethical committees from Complutense University and Madrid region according to European regulations.

## Results

### MDK regulates the self-renewal capacity of GICs

**Figure 1:**
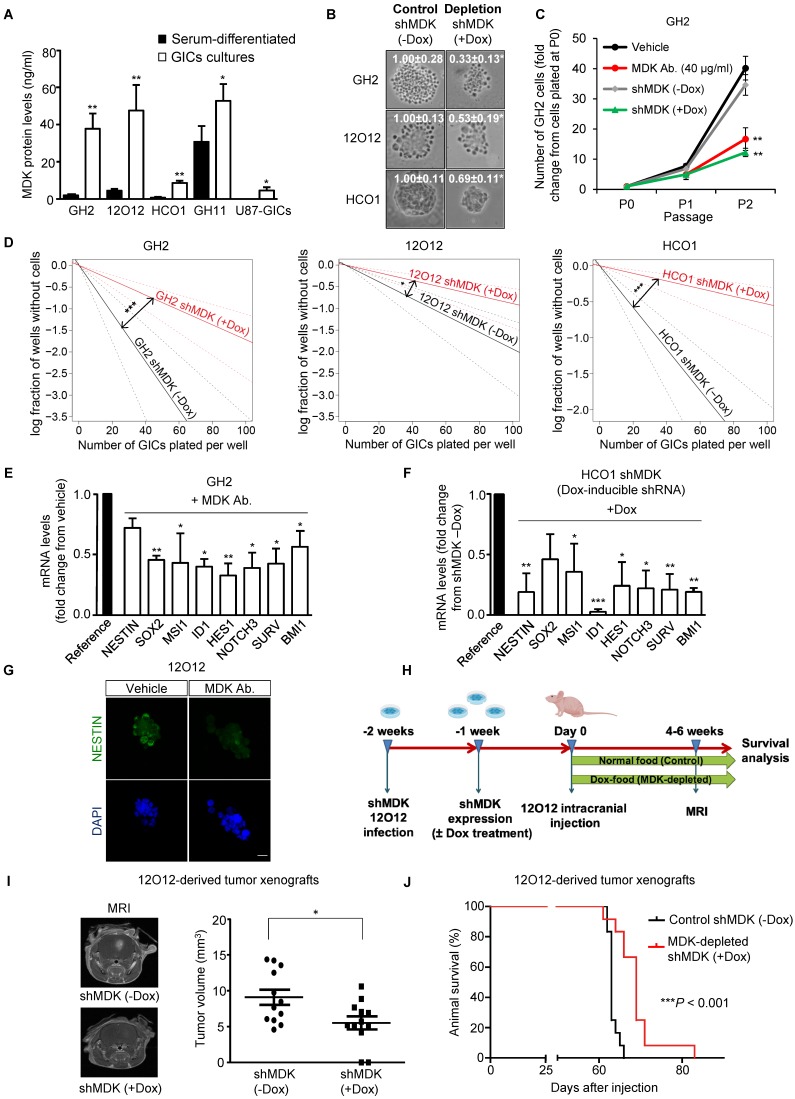
** MDK regulates the self-renewal capacity and tumorigenic properties of GICs. (A)** MDK protein levels (as determined by ELISA) in the medium of GICs cultures or of their corresponding serum-differentiated cells. Data correspond to MDK concentration (ng/ml) and are expressed as mean concentration ± SEM (n=3). *P < 0.05; **P < 0.01 from serum-differentiated cells. **(B)** Effect of MDK genetic inhibition [by treating with doxycycline (+Dox) cells stably transduced with a doxycycline-inducible MDK-selective (shMDK) shRNA] on the morphology of GH2, 12O12 and HCO1-GICs cultures. Representative images obtained by phase-contrast microscopy are shown. Values in each image correspond to the neurosphere's area (nm^2^) and are expressed as mean fold change ± SEM. At least 4-5 neurospheres were analyzed for each experimental condition in each GIC culture. *P < 0.05 from shMDK (-Dox). **(C)** Effect of MDK genetic inhibition or incubation with an anti-MDK antibody (MDK Ab., 40 μg/ml) on the growth of GH2-GICs (n=3). **P < 0.01 from vehicle or shMDK (-Dox) cells. **(D)** Effect of MDK genetic inhibition (+Dox) on the self-renewal ability (as determined by LDA) of GH2, 12O12 and HCO1-GICs (n=2). *P < 0.05; ***P < 0.001 from shMDK (-Dox). **(E-F)** Effect of MDK depletion by incubation with an anti-MDK antibody (MDK Ab., 40 μg/ml, 72h) (GH2-GICs, panel E) or by expressing a doxycycline-inducible shMDK (+Dox; HCO1-GICs, panel F) on the mRNA levels of a panel of stem cell associated genes. Data are expressed as mean fold change from vehicle (panel E) or shMDK (-Dox) (panel F)-treated cells (reference) ± SEM (n=3). *P < 0.05; **P < 0.01; ***P < 0.001 from vehicle-treated cells (panel E) or from shMDK (-Dox) (panel F). **(G)** Effect of the incubation with an anti-MDK antibody (MDK Ab., 40 μg/ml, 72 h) on NESTIN expression (as determined by immunostaining) of 12O12-GICs. Scale bar: 20 μm. **(H)** Procedure to generate intracranial xenografts with 12O12 shMDK-GICs. **(I)** Effect of MDK genetic inhibition [shMDK (+Dox)] on the size of glioma xenografts generated by intracranial injection of 7.5 x 10^4^ 12O12 shMDK-GICs (n=12). Representative MRI images 6 weeks after the injection are shown (left panel). Tumor volume is expressed as mean ± SEM (right panel). *P < 0.05 from 12O12 shMDK (-Dox) tumors. **(J)** Effect of MDK genetic inhibition [shMDK (+Dox)] on the survival of tumor-bearing mice. Kaplan-Meier plot (n=12). ***P < 0.001 from 12O12 shMDK (-Dox) tumors.

Analysis of GBM patient's datasets from published studies revealed the existence of a clear correlation between high MDK expression and worse prognosis in GBM patients (Figure S1A-F). Therefore, we asked whether MDK may play an oncogenic role in GBM via a positive regulation of GICs. We used a panel of GICs cultures derived from GBM samples, which were extensively characterized *in vitro* to confirm the expression of bona-fide (glioma) stem cell markers (Supplementary Table 1 and Figure S2A-B). MDK protein levels present in the medium (Figure 1A and Figure S2C) as well as MDK mRNA levels (Figure S2D) were dramatically increased in GICs-enriched cultures (hereafter named GICs cultures) as compared with the levels of this neurotrophic factor in the corresponding cultures of serum-differentiated glioma cells. Moreover, genetic inhibition of MDK by using a doxycycline-inducible MDK-selective shRNA (Figure S2E-F) [note that controls of silencing of this and other panels are included in the supplementary Figures], reduced the size of the neurospheres generated with GICs (Figure 1B). Likewise, genetic inhibition of MDK or removal of MDK from the medium by using an anti-MDK neutralizing antibody (Figure S2G) strikingly reduced the capacity of GICs to generate new neurosphere cultures during two consecutive passages (Figure 1C and Figure S2H) and inhibited the self-renewal capacity, as determined by the limited dilution assay [LDA [20]](Figure 1D and Figure S2I).

The observed reduction in GIC neurosphere generation in the absence of MDK correlated with a striking reduction in the expression of a panel of stem cell markers (Figure 1E-G and Figure S3A-F). Specifically, we verified that MDK depletion decreased the number of NESTIN and MUSASHI (MSl1) (two well-established biomarkers of stemness 6, 29)-positive cells in neuro-sphere cultures (Figure 1G and S3D-E). Likewise, abrogation of MDK signaling markedly diminished protein levels of these two stem cell markers (Figure S3E).

To investigate the role of MDK in the regulation of the tumorigenic capacity of GICs *in vivo*, we injected 12O12 GICs expressing a doxycycline- inducible MDK-selective shRNA into the striatum of immunodeficient mice (Figure 1H). Doxycycline- induced knock-down of MDK expression (Figure S3G) delayed the onset (Figure S3H) and inhibited the growth (Figure 1I) of 12O12 GICs-derived orthotopic xenografts. Likewise, MDK genetic depletion enhanced the survival of the mice bearing tumors generated with these cells (Figure 1J and Figure S3I-L).

### MDK effects on GICs rely on ALK stimulation

**Figure 2:**
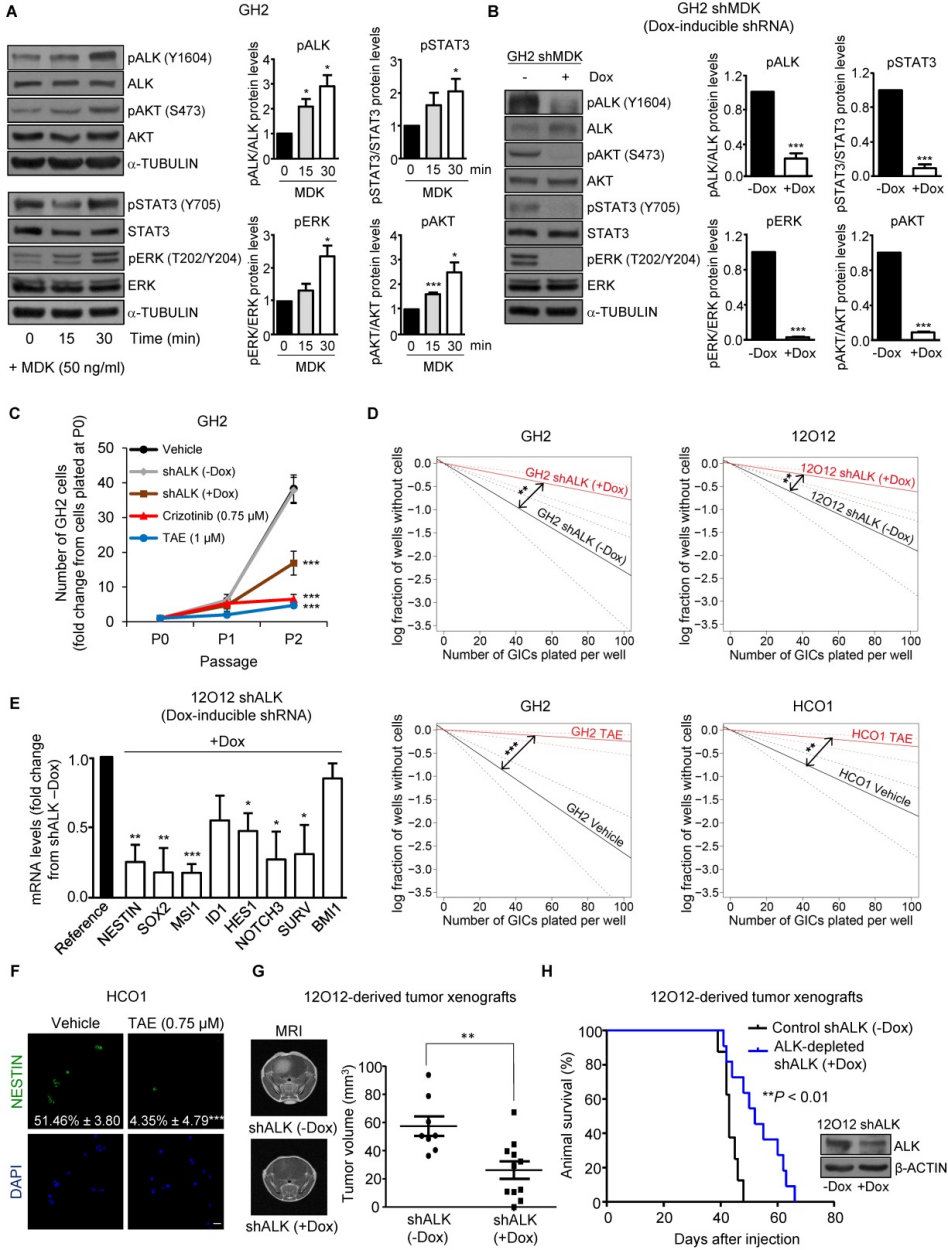
** MDK/ALK axis plays a relevant role in the maintenance of the stem-like properties and the tumorigenic capacity of GICs. (A)** Effect of the incubation with exogenous MDK (MDK, 50 ng/ml) on the phosphorylation of ALK (pALK), AKT (pAKT), ERK (pERK) and STAT3 (pSTAT3) of GH2-GICs at different time points. Left panel: a representative Western blot is shown (n=3). Right panel: Data correspond to the densitometric analysis of the levels of each phosphorylated protein relative to total levels of that protein and are expressed as the mean fold change ± SEM relative to vehicle-treated cells (n=3). *P < 0.05 and ***P < 0.001 from MDK 0 min-treated cells. **(B)** Effect of MDK genetic inhibition [by treating with doxycycline (+Dox) cells stably transduced with a doxycycline-inducible MDK-selective (shMDK) shRNA] on the phosphorylation of ALK (pALK), AKT (pAKT), ERK (pERK), and STAT3 (pSTAT3) of GH2-GICs. Left panel: a representative Western blot is shown (n=3). Right panel: data correspond to the densitometric analysis of the levels of each phosphorylated protein relative to total levels of that protein and are expressed as the mean fold change ± SEM relative to shMDK (-Dox)-treated cells (n=3). ***P < 0.001 from (-Dox)-treated cells. **(C)** Effect of ALK genetic inhibition (by treating with doxycycline (+Dox) cells stably transduced with a doxycycline-inducible shALK) or incubation with TAE (1 μM) or crizotinib (0.75 μM) on the growth of GH2-GICs (n=3). ***P < 0.001 from vehicle or shALK (-Dox) cells. **(D)** Effect of ALK genetic inhibition (+Dox) or incubation with TAE (0.75 μM) on the self-renewal ability (as determined by LDA) of GH2, 12O12 and HCO1-GICs (n=2). **P < 0.01 from shALK (-Dox.) cells or HCO1 vehicle-treated cells; ***P < 0.001 from GH2 vehicle-treated cells. **(E)** Effect of ALK genetic inhibition (by treating with doxycycline (+Dox) cells stably transduced with a doxycycline-inducible shALK) on the mRNA levels (as determined by qPCR) of a panel of stem cell associated genes in 12O12 shALK-GICs. Data are expressed as mean fold change from shALK (-Dox)-treated cells (reference) ± SEM (n=3). *P < 0.05; **P < 0.01; ***P < 0.001 from 12O12shALK (-Dox). **(F)** Effect of the incubation with TAE (0.75 µM, 72 h) on NESTIN expression (as determined by immunofluorescence) of HCO1-GICs. Values in each photomicrograph correspond to the percentage of NESTIN-positive cells relative to the total number of nuclei. Representative photomicrographs are shown (n=3). ***P < 0.001 from vehicle-treated cells. Scale bar: 20 μm. **(G)** Effect of ALK genetic inhibition on the size of glioma xenografts (MRI) generated by intracranial injection of 7.5 x 10^4^ 12O12 shALK-GICs. Representative MRI images 6 weeks after the injection are shown (left panel). Tumor volume is expressed as mean ± SEM (right panel) (n=8-11). **P < 0.01 from 12O12 shALK (-Dox) tumors. **(H)** Effect of ALK genetic inhibition on the survival of tumor-bearing mice. Kaplan-Meier plot (n=12). ***P < 0.001 from 12O12 shALK (-Dox) tumors. Inset: Effect of doxycycline (+Dox) on ALK protein levels of 12O12 shALK cultures determined right before their intracranial injection.

The ALK receptor tyrosine kinase (ALK) has been proposed to mediate MDK actions in cancer cells 10, 12, 30. We found that ALK protein levels were increased in GICs when compared with their serum-differentiated counterparts (Figure S4A). In addition, MDK treatment increased the phosphorylation of ALK as well as of some of its well-established downstream targets (STAT3, ERK and AKT)^31^, ^32^ in GH2 GICs (Figure 2A), and this effect was prevented by ALK pharmacological inhibition using NVP-TAE 684 (TAE 33) (Figure S4B). Likewise, MDK silencing decreased the phosphorylation of ALK and its downstream targets (Figure 2B), supporting the idea that MDK effects in GICs rely on ALK stimulation. In agreement with this hypothesis, we found that the genetic inhibition of ALK or the pharmacological blockade of this receptor using TAE as well as crizotinib and lorlatinib (two drugs that are currently being used in clinical oncology to target ALK)^34^ reduced the ability to form neurospheres during two consecutive passages (Figure 2C and Figure S4C) and inhibited the self-renewal capacity (Figure 2D and Figure S4D) of three different cultures of GICs. This decrease in the stem-like properties of GICs was consistent with a downregulation of the expression of a panel of stem cell markers upon ALK inhibition (Figure 2E and Figure S4E-F). Specifically, ALK pharmacological blockade decreased the number of NESTIN (Figure 2F) and MSI1 (Figure S4G)-positive cells, supporting the notion that the MDK-ALK axis is involved in the regulation of the stem cell properties of GICs. In line with this idea, doxycycline-induced ALK silencing inhibited tumor growth and increased the survival of animals bearing GICs-derived intracranial xenografts (Figure 2G-H).

### MDK/ALK inhibition-induced effects on GICs rely on SOX9 degradation

**Figure 3:**
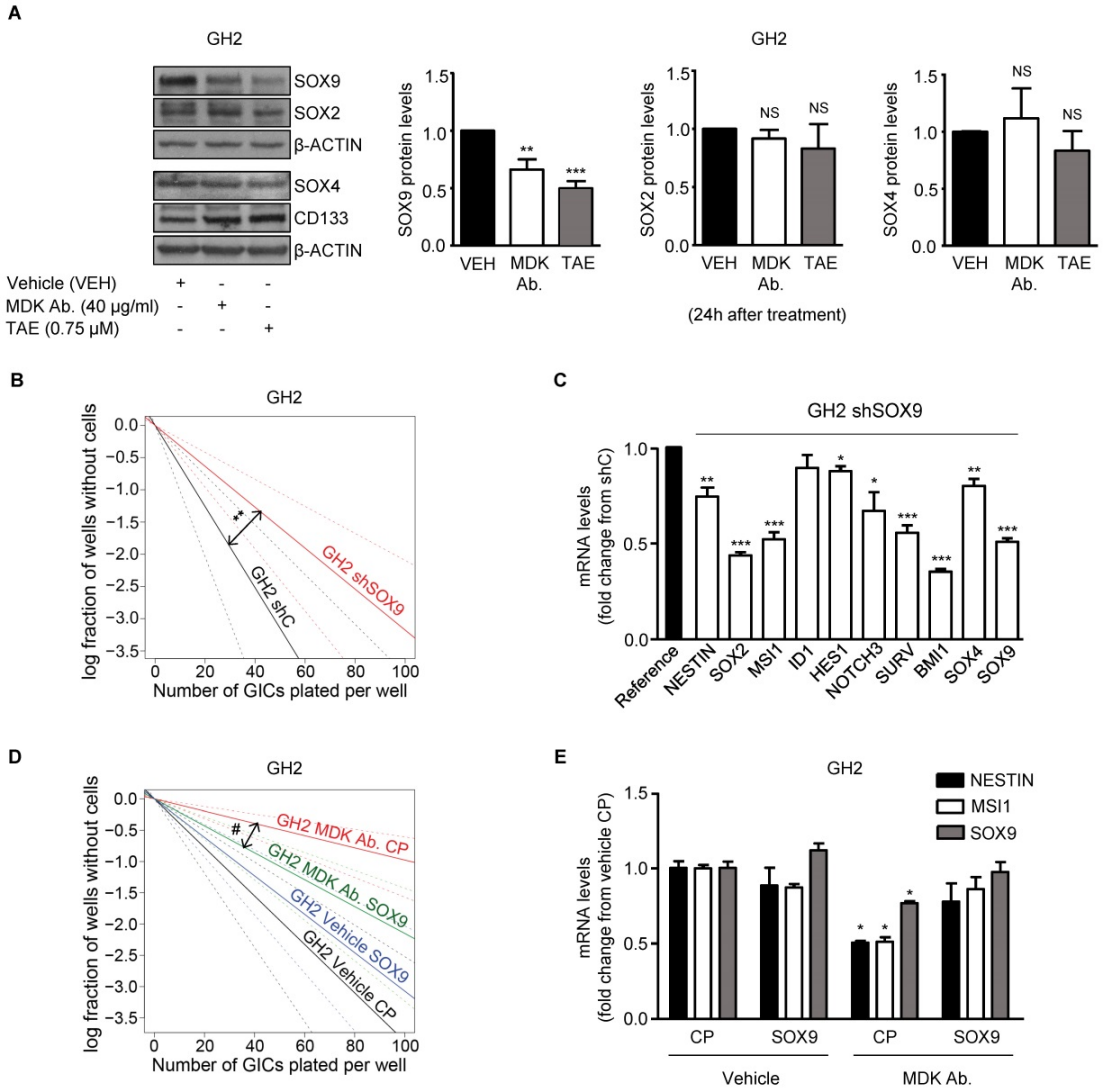
** MDK/ALK signaling axis regulates the maintenance of the stem-like properties of GICs by controlling SOX9 protein levels. (A)** Effect of the incubation with an anti-MDK antibody (MDK Ab., 40 μg/ml) or TAE (0.75 μM) for 24 h on SOX2, SOX4, SOX9 and CD133 protein levels of GH2-GICs. Left panel: a representative Western blot experiment is shown (n=5). Right panel: densitometric analysis of SOX9, SOX2 and SOX4 protein levels (mean fold change from vehicle ± SEM; n=5 for SOX9 and SOX2 and n=3 for SOX4). **P < 0.01 and ***P < 0.001 from vehicle-treated cells. NS: statistically non-significant differences. **(B)** Effect of SOX9 genetic inhibition [by nucleofection with a plasmid encoding a shcontrol (shC) or a SOX9-selective (shSOX9) shRNA] on the self-renewal ability (as determined by LDA) of GH2-GICs (n=3) **P < 0.01 from shC cells. **(C)** Effect of SOX9 genetic inhibition (72 h) on mRNA levels of a panel of stem cell associated genes (as determined by qPCR) of GH2-GICs. Data are expressed as mean fold change from shC cells (reference) ± SEM (n=3). *P < 0.05; **P < 0.01; **P < 0.001 from shC cells. **(D)** Effect of the incubation with an anti-MDK antibody (MDK Ab., 40 μg/ml) and nucleofection with a control plasmid (CP), or a plasmid encoding a murine SOX9 (SOX9) on the self-renewal ability (as determined by LDA) of GH2-GICs (n=2). ^#^*P* < 0.05 from MDK Ab.-treated CP cells. Full χ^2^ statistical analysis is included in LDA statistics section within the supplementary materials. **(E)** Effect of the incubation with an anti-MDK antibody (MDK Ab., 40 µg/ml) on the mRNA levels of *NESTIN*, *MUSASHI-1 (MSI1)* and *SOX9* (as determined by qPCR) of GH2-GICs nucleofected with a control plasmid (CP) or a plasmid encoding a murine SOX9 (SOX9) (72 h). *P < 0.05 from vehicle CP-treated cells.

The SOX family of transcription factors plays a pivotal role in the regulation of the maintenance of the stem-like properties of GICs 35. Accordingly, we found that blockade of the MDK/ALK axis in GICs cultures triggered a sustained downregulation of several stem cell markers including various SOXs, which correlated with the observed decrease in the self-renewal capacity of GICs (Figure 1E, 1F, 2E and Figure S5A-B). Specifically, we found that MDK/ALK inhibition for 72 h or longer timepoints led to decreased expression (associated with a transcriptional regulation) of several SOX family members with oncogenic activity including *SOX2*, *SOX4* and *SOX9* (Figure S5A-B). In addition, analysis of SOXs protein levels at early time points (24 h after the blockade of the MDK/ALK axis, a time point which changes in the transcriptional regulation of these genes were not observed yet) showed a consistent reduction of SOX9 but not of SOX2 or SOX4 or of other stem cell markers in GICs cultures (Figure 3A, Figure S5C). In agreement with the idea that SOX9 plays a key regulatory role in the triggering of the events that lead to the loss of the stem-like properties of GICs upon blockade of the MDK/ALK axis, SOX9 silencing mimicked the effect of MDK/ALK axis inhibition in GICs and resulted in a lower efficiency of neuro-sphere formation (Figure 3B and Figure S5D) as well as in a clear reduction in the mRNA levels of several stem cell markers, including *SOX2* and *SOX4* (Figure 3C and Figure S5E). Conversely, re-expression of a murine form of SOX9 in GICs cultures (Figure S5F) prevented the inhibition of the self-renewal capacity triggered by the genetic (Figure S5G) or pharmacological (Figure 3D) inhibition of MDK, as well as the decrease in the expression of several stem cell markers evoked by incubation with an anti-MDK neutralizing antibody (Figure 3E). Likewise, MDK/ALK inhibition decreased the expression levels of c-MYC and CYCLIND1, two well-stablished SOX9 targets (Figure S5H). These observations strongly support the idea that the MDK/ALK signaling axis regulates the maintenance of the stem-like properties of GICs by controlling SOX9 protein levels.

SOX9 levels have been shown to be regulated transcriptionally and by the poly-ubiquitylation and subsequent degradation of this protein in the proteasome 35, 36. However, MDK/ALK axis inhibition did not modify SOX9 mRNA levels at 24 h (the timepoint at which the initial reduction of SOX9 protein levels was observed) (Figure S6A). Likewise, pharmacological blockade of the proteasome by lactacystin did not prevent the decrease of SOX9 protein levels triggered by the blockade of the MDK/ALK axis (Figure S6B) suggesting that these mechanisms are not responsible for SOX9 stability in this model.

### Blockade of the MDK/ALK axis induces autophagy in GICs

ALK is coupled to the activation of the AKT/ MTORC1 pathway 30, 31 and we found that MDK/ ALK axis inhibition decreased the phosphorylation of AKT (Figure 2A, 2B and S4B), of TSC2 (a well stablished AKT downstream target that when phosphorylated contributes to stimulate the activity of the MTORC1 complex) as well as of the ribosomal protein S6 (Figure S6C), a direct MTORC1 target. Since inhibition of the MTORC1 axis triggers autophagy and this process is one of the main cellular mechanisms responsible for protein degradation 37, we asked if the inhibition of the MDK/ALK axis stimulated autophagy in GICs.

**Figure 4:**
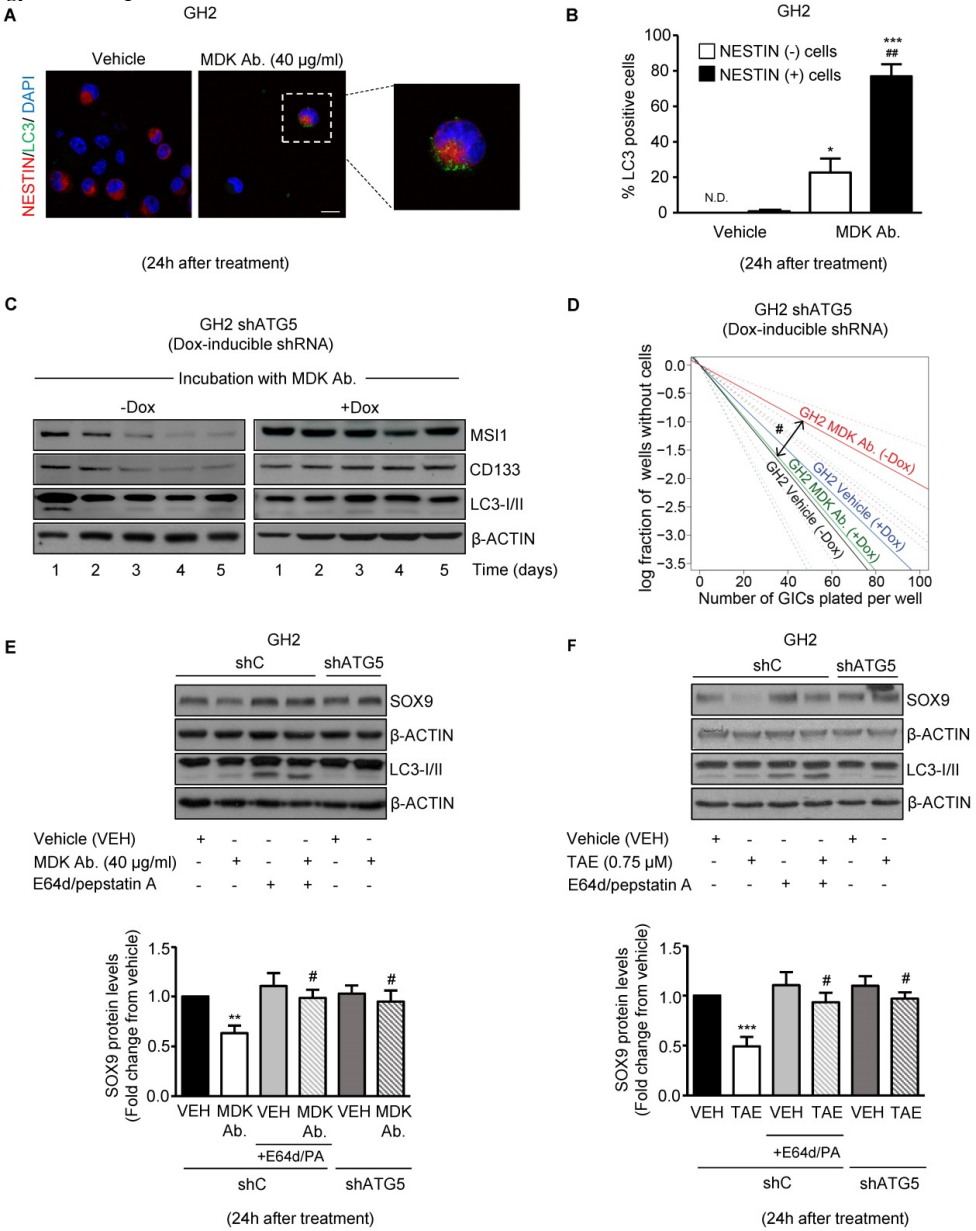
** Blockade of the MDK/ALK axis in GICs induces the autophagic degradation of SOX9. (A)** LC3 and NESTIN immunostaining of GH2-GICs cells incubated with an anti-MDK antibody (MDK Ab., 40 μg/ml, 24 h) (n=3). Representative images (with a high magnification photomicrograph of the squared-pointed area of MDK Ab.-treated cells) are shown. Scale bar: 20 μm. **(B)** Quantification of the percentage of cells with LC3 dots within the population of NESTIN-positive or negative GH2-GICs (n=3). *P < 0.05 ***P < 0.001 from vehicle-treated cells; ##*P* < 0.01 from MDK Ab.-treated NESTIN-negative cells. ND: Non-detectable. **(C)** Effect of the incubation with an anti-MDK antibody (MDK Ab., 40 μg/ml) on MUSASHI1 (MSI1), CD133 and LC3-I/II protein levels of GH2-GICs stably transduced with a doxycycline-inducible shATG5 at different time points (n=3). A representative experiment is shown. **(D)** Effect of the incubation with an anti-MDK antibody (MDK Ab., 40 µg/ml) and the genetic inhibition of autophagy on the self-renewal ability (as determined by LDA) of GH2-GICs (n=2). ^#^*P* < 0.05 from GH2 shATG5 (-Dox.) MDK Ab.-treated cells. Full χ^2^ statistical analysis is included in LDA statistics section within the supplementary materials. **(E-F)** Effect of the incubation with an anti-MDK antibody (MDK Ab., 40 μg/ml; 24 h, panel E) or TAE (0.75 μM, 24 h, panel F) on SOX9 and LC3-I/II protein levels of shC or shATG5-transduced GH2-GICs cultures untreated or pretreated with E64d (10 μM) and pepstatin A (PA, 10 μg/ml) for 1 h. A representative Western blot (upper panels) and the corresponding densitometric quantifications (bottom panels) are shown (n=5). **P < 0.01 from vehicle-treated cells; ^#^*P* < 0.05 from MDK Ab.-treated shC cells.

In support of this hypothesis, blockade of MDK/ALK signaling led to a consistent increase in autophagy at 24 h, as determined by the accumulation of the autophagosome-associated form of the autophagy-related protein LC3 (LC3-II, which exhibits a higher electrophoretic mobility than LC3-I, the form of the protein not associated to autophagosomes 37) (Figure S6D) and by the accumulation of LC3 dots in the cytoplasm of GICs (Figure S6E), two hallmarks of autophagy 37. Furthermore, we found that pharmacological inhibition of lysosomal proteases (by using E64d and pepstatin A) in combination with MDK/ALK inhibitors led to a further accumulation of LC3-II (Figure S6D) in GICs cultures, thereby confirming that blockade of the MDK/ALK axis induces dynamic autophagy in these cells. Moreover, accumulation of LC3 dots upon incubation with an anti-MDK antibody for 24 h occurred preferentially in NESTIN-positive cells (Figure 4A and 4B) indicating that the stimulation of autophagy observed upon inhibition of MDK signaling took place in the population of cells exhibiting a stem-like phenotype.

### Autophagy is required for the loss of the stem-like phenotype evoked by the blockade of MDK/ALK axis in GICs

To analyze the relevance of the early stimulation of autophagy in the loss of stem-like properties observed in GICs upon blockade of MDK/ALK signaling, we analyzed the effect of MDK signaling inhibition in GICs cultures expressing a doxycycline- inducible shRNA against the autophagy essential protein ATG5 37 (Figure S7A). As shown in Figure 4C and Figure S7B, MDK neutralization triggered an early (24 h) and transient induction of autophagy followed by a sustained reduction in the expression of the stem-cell markers MSI1 and CD133 in autophagy- proficient GH2-GICs but not in ATG5-silenced GH2-GICs. Moreover, genetic inhibition of autophagy by using the above-described doxycycline- inducible shRNA against ATG5 (Figure 4D and Figure S7C) or a constitutive shRNA against ATG7 (another essential autophagy gene; Figure S7D-E) abrogated the inhibitory effect triggered by MDK/ALK pharmacological inhibition on the self-renewal capacity of GICs cultures. Altogether, these results strongly support the notion that stimulation of autophagy is required for the loss of the stem-like phenotype evoked by the blockade of MDK/ALK axis in GICs.

We next investigated whether MDK/ALK axis blockade-triggered autophagy stimulation is responsible for SOX9 degradation in GICs. In line with this idea, pharmacological inhibition of protein degradation through the autophagosome-lysosome pathway by using E64d + pepstatin A, or genetic inhibition of autophagy by knocking down the expression of ATG5 (Figure S7F), prevented the decrease of SOX9 protein levels triggered by incubation with an anti-MDK neutralizing antibody (Figure 4E) or by the pharmacological inhibition of ALK (Figure 4F). Similar results were obtained by knocking down the expression of ATG7 (Figure S7G). These findings demonstrate that the blockade of the MDK/ALK axis in GICs triggers SOX9 degradation via autophagy stimulation.

It has been proposed that the targeting and subsequent degradation of protein cargos through the autophagosome-lysosome pathway involves the unspecific incorporation of cytoplasmic or organelle- associated proteins into the autophagosome 38. In addition, at least a fraction of cellular proteins can be selectively targeted to the autophagosomes - via direct interaction with LC3 or other proteins involved in the formation of the phagophore (the initial structure that gives origin to the autophagosomal membrane) or via cargo-transporting proteins 37, 38. Therefore, next we asked whether the autophagic degradation of SOX9 observed in GICs upon blockade of the MDK/ALK axis was due to the selective targeting of this transcription factor to the autophagosome. As shown in Supplementary Figure 7H, immunoprecipitation of LC3 from vehicle-treated GH2-GICs pulled down SOX9 and this effect was enhanced when lysosomal proteases were inhibited, suggesting that regulation of SOX9 levels relies on its association with the autophagic machinery and further supporting the idea that autophagy is involved in the regulation of SOX9 levels in GICs. In addition, the interaction of SOX9 with the LC3 complex was abolished upon blockade of the MDK/ALK axis and the subsequent induction of SOX9 autophagic degradation (Figure S7H), whereas pharmacological inhibition of the lysosomal proteases prevented SOX9 degradation and restored the interaction between SOX9 and the LC3 complex. Likewise, pharmacological blockade of lysosomal degradation enhanced the interaction of immunoprecipitated SOX9 with the pulled down autophagosome-associated form of LC3 upon MDK/ ALK inhibition (Figure S7I). Taken together, these observations demonstrate that the blockade of the MDK/ALK axis in GICs stimulates SOX9 degradation through the autophagy-lysosome pathway.

### Blockade of the MDK/ALK axis targets GICs *in vivo*

**Figure 5:**
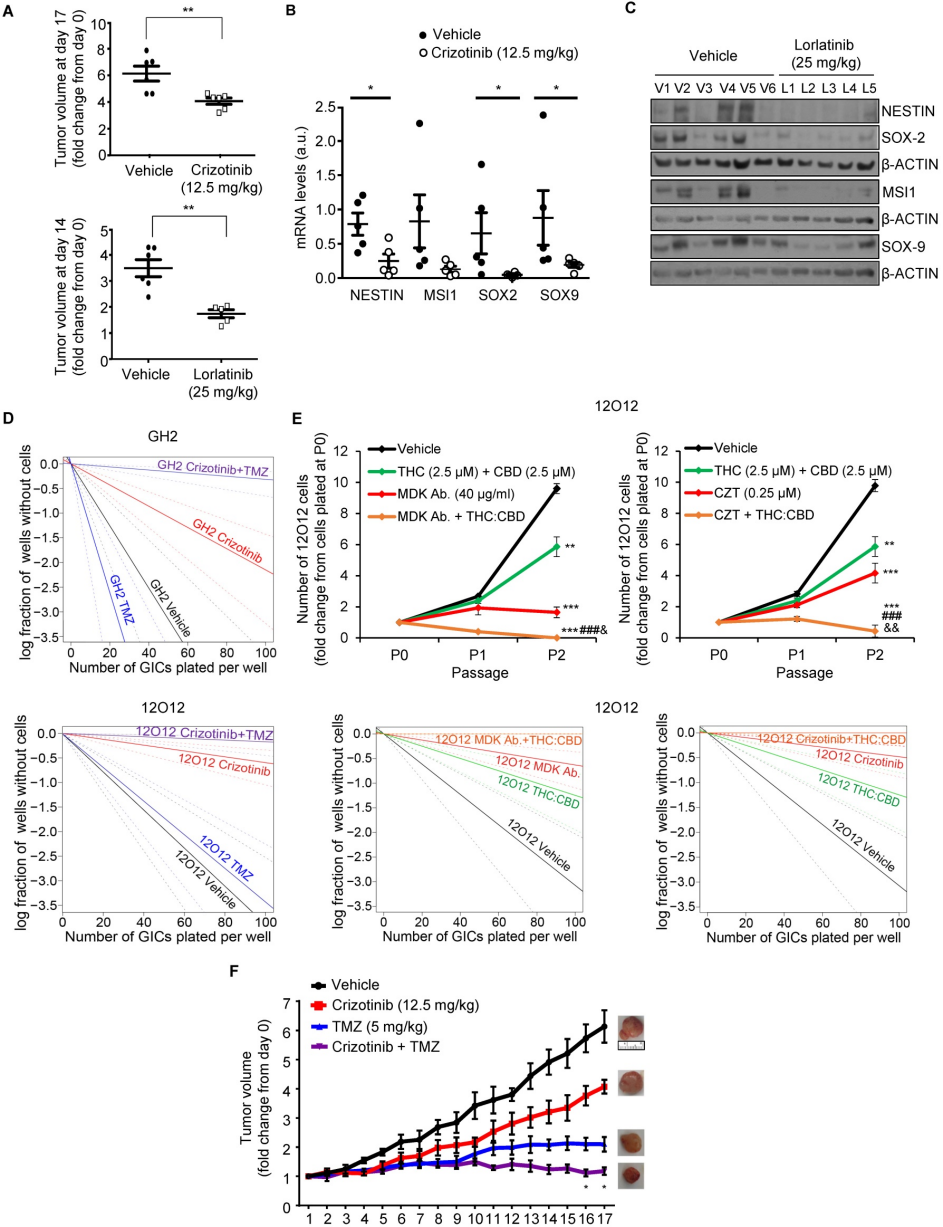
** Combination of MDK/ALK inhibitors with cannabinoids and TMZ strongly reduces the growth of GICs-derived xenografts. (A)** Effect of daily oral administration of crizotinib (12.5 mg/kg, upper panel) or lorlatinib (25 mg/kg, bottom panel) on the volume of glioma xenografts generated by subcutaneous injection of 2 x 10^6^ 12O12-GICs (mean ± SEM on the last day of the treatment). **P < 0.01 from 12O12 vehicle-treated tumors. **(B)** Effect of daily oral administration of crizotinib (12.5 mg/kg) on the mRNA levels of *NESTIN*, *MUSASHI-1* (*MSI1*), *SOX2* and *SOX9* (as determined by qPCR) of glioma xenografts generated by subcutaneous injection of 2 x 10^6^ 12O12-GICs (mean ± SEM). *P < 0.05 from 12O12 vehicle-treated tumors. **(C)** Effect of daily oral administration of lorlatinib (25 mg/kg) on the protein levels (as determined by Western blot) of NESTIN, MUSASHI-1 (MSI1), SOX2 and SOX9 in the tumor xenografts (V1-V6: vehicle-treated animals; L1-L5: lorlatinib-treated animals). **(D)** Effect of the treatment with crizotinib (0.5 µM) and TMZ (100 μM, upper panel or 20 μM bottom panel) on the self-renewal ability (as determined by LDA) of GH2 (upper panel) or 12O12 (bottom panel)-GICs (n=2). Full χ^2^ statistical analysis is included in LDA statistics section within the supplementary materials. **(E)** Effect of the treatment with THC:CBD (2.5 μM THC + 2.5 μM CBD) and MDK Ab. (40 μg/ml, left panel) or crizotinib (CZT, 0.25 µM, right panel) on the total number of cells (upper panels) and self-renewal capacity (bottom panels) of 12O12-GICs (n=3). **P < 0.01 and ***P < 0.001 from vehicle-treated cells; ^###^*P* < 0.001 from THC + CBD-treated cells; ^&^*P* < 0.05 or^ &&^*P* < 0.01 from MDK Ab.-treated cells or crizotinib-treated cells. Full χ^2^ statistical analysis is included in LDA statistics section within the supplementary materials. **(F)** Effect of the treatment with crizotinib (12.5 mg/kg daily oral administration) and TMZ (5 mg/kg twice a week IP administration) on the growth of glioma xenografts generated by subcutaneous injection of 2 x 10^6^ 12O12-GICs [mean ± SEM; n=5-6 mice for each condition]. Representative pictures of the tumor xenografts in the last day of the treatment are shown for each experimental condition. Symbols of significance are omitted for clarity except when the combined-treatment was significantly different from vehicle and each individual treatment (*P < 0.05 or **P <0.01 from each individual treatment). The rest of the statistical analysis can be found in Supplementary materials.

To investigate whether blockade of the MDK/ALK axis may be used as a strategy to target the population of GICs *in vivo*, we tested the effect of some of the ALK inhibitors that are currently being used in anticancer therapies. As described above, blockade of ALK using crizotinib inhibited the self-renewal capacity of GICs cultures (Figure 2C, Figure S4C-D), reduced the expression of several stem cell markers (Figure S4E-F) and induced autophagy (Figure S6D-E) in cultures of these cells. Similar results were obtained with lorlatinib (Figure S4C and S8A). Accordingly, treatment with crizotinib or lorlatinib alone strikingly reduced the growth of GICs-derived tumors (Figure 5A). Moreover, treatment with these agents produced a strong reduction in the expression of stem cell markers (Figure 5B and 5C and Figure S8B-C), supporting the idea that ALK pharmacological inhibition reduces the population of GICs *in vivo*.

### Combination therapies based on the blockade of the MDK/ALK axis as a strategy to eliminate the population of GICs

Current strategies to treat GBM are based on the use of combination therapies. Therefore, we next asked whether the administration of inhibitors of the MDK/ALK axis may contribute to improve the efficacy of current anti-GBM treatments. To test this hypothesis, we initially selected temozolomide (TMZ, the benchmark agent for the management of GBM) as the effect of the combination of crizotinib and TMZ on GBM patients is currently being tested in a clinical study (clinical trials.gov identifier: NCT02270034). The inhibitory effect of crizotinib on the self-renewal capacity of GICs was dramatically enhanced by the co-administration of TMZ (Figure 5D) leading to an almost complete elimination of the population of GICs. Likewise, apoptosis was strikingly activated in GICs cultures upon the combined administration of these two agents (Figure S9A). Similar results were obtained when we tested the combined effect of MDK/ALK axis inhibitors and cannabinoids, a novel family of potential anticancer agents 39, 40 that have been shown to synergize with ALK inhibitors in differentiated glioma cells 13, 39, 40. The combined administration of submaximal doses of crizotinib or of an anti-MDK neutralizing antibody and Δ^9^-tetrahydrocannabinol (THC, the main active ingredient of marijuana) strongly enhanced the effect of the individual treatments in the self-renewal capacity of GICs, leading to an almost complete elimination of the population of GICs *in vitro* (Figure S9B-C). Likewise, the combined administration of MDK/ALK axis inhibitors together with a mixture of THC and cannabidiol (CBD, another active ingredient of Cannabis sativa) at a 1:1 ratio exerted a similar inhibitory effect on the generation of neurospheres and the self-renewal capacity of GICs (Figure 5E). Furthermore, we found that the combined administration of crizotinib, or an anti-MDK neutralizing antibody and THC, but not treatment with these agents individually, triggered apoptosis and GICs death (Figure S9D).

Therefore, next we tested the effect of the combined administration of these agents in xenografts generated with GICs. Treatment with crizotinib and TMZ (Figure 5F) produced a much stronger reduction on the growth of xenografts generated with 12O12-GICs than the treatments with each of those agents alone. Likewise, apoptosis was activated to a much higher extent in samples derived from tumors that had received the combined treatments than in those that had been treated with crizotinib or TMZ alone (Figure S9E). Furthermore, a similar enhanced anticancer activity was observed when lorlatininb was combined with TMZ (Figure S9F).

Altogether, these findings strongly support the notion that MDK/ALK axis inhibition-based combinational therapies can be used as a strategy to deplete the population of GICs and improve the efficacy of current anti-GBM therapies.

## Discussion

Work performed during the last decades has unraveled the importance of “cancer stem-like cells” in the generation and progression of many different cancers. In the case of GBM, the very high frequency of relapses and resistance to conventional therapies typical of these tumors has led to postulate that GICs play a crucial role in their aggressive evolution. GBMs are highly heterogeneous and contain cells with different degrees of differentiation, being the subpopulation of GICs a very minor proportion of cells within the tumor mass 6, 7. Thus, the stimulatory effect evoked by different growth factors on GICs seems to be of crucial importance to preserve their self-renewal capacity and therefore for the maintenance of this sub-population within the tumor mass 6, 7, 41, 42. Results presented in this study now demonstrate that the neurotrophic factor MDK plays a direct role in the regulation of the self-renewal and tumorigenic capacity of GICs, supporting the notion that increased levels of this factor decisively contribute to maintain this population. In line with this idea, increased levels of MDK correlate with lower survival of GBM patients which suggests that the upregulation of this factor may contribute to enhance the aggressiveness of GBMs by maintaining/ expanding the population of GICs. In addition, previous observations had shown that enhanced MDK expression in differentiated glioma cells promotes resistance to the treatment with cannabinoids and other anticancer agents 12, 13. Of note, recent results have shown that MDK plays a paracrine role in the regulation of lymphatic endothelial cells during melanoma metastasis 43.

Likewise, it was recently reported that p53-dependent MDK-induction upon DNA damage in gliomas promoted M2 polarization of microglia and that this event would play a relevant role on the remodeling of the tumor microenvironment 44. Taken together, these observations suggest that part of the oncogenic effects of MDK in GBM may also be due to the capacity of this factor to interact with the stroma. Moreover, altogether these observations point to the potential utilization of MDK as a biomarker of bad prognosis and resistance to therapies in GBM.

Our findings also show that MDK effects on GICs rely on ALK stimulation. These observations are in line with previous work by our group showing that MDK expression promoted resistance to anticancer agent-induced differentiated glioma cell death via this tyrosine kinase receptor rather than PTPRz or other receptors that have been proposed to mediate MDK actions in other contexts 10. Nevertheless, it cannot be discarded that some of these receptors may also contribute - at least to some extent - to MDK actions in gliomas or GICs.

ALK has been shown to undergo gain of function mutations in different cancer types including non-small-cell lung cancer, anaplastic large cell lymphomas and neuroblastoma although not - or with a very low frequency - in gliomas 32. Results presented here, together with previous observations obtained by other groups 45 indicate that enhanced ligand-triggered ALK stimulation rather than genetic alterations on this receptor would be responsible for the effects observed in GICs and therefore in GBM biology.

Results from the current study also demonstrate that the mechanism by which the MDK/ALK axis regulates GICs biology is based on the control of the stability of SOX9. The SOX family of transcription factors plays a crucial role in the regulation of stem-cell biology 35, 46. Results obtained in this work are in line with the idea that these transcription factors, and specifically SOX9, are essential for the maintenance of the stem-like properties and self-renewal capacity of GICs. Our findings reveal that the autophagosome-lysosome pathway rather than other mechanisms that have been previously shown to be involved in regulating SOX9 levels 46 is responsible for the control of SOX9 stability in GICs. Thus, the blockade of the signal evoked by MDK/ALK in GICs triggers a transient activation of autophagy that is responsible for the selective degradation of SOX9 rather than of other SOX transcription factors. Whether MDK/ALK signaling controls the stem like properties of GICs via the regulation of a specific subset of SOX9-regulated genes is an interesting possibility that remains to be investigated.

Depending on the stage of the tumor and cancer type, autophagy has been shown to play oncogenic or onco-suppressor functions 47, 48. Our data support the idea that MDK/ALK signaling contributes to GICs maintenance by suppressing the autophagic degradation of SOX9. It is worth noting that, although autophagy has been proposed to play a supportive role in cancer stem cells 47, 49 including GBM 50, we found that genetic or pharmacological blockade of autophagy does by itself significant alter the self- renewal capacity of GICs cultures. These apparently contradictory observations may be reconciled in a model in which the selectivity of the cargos targeted for degradation to the autophagosome-lysosome pathway or the intensity/duration with which this cellular process is stimulated determine the role and outcome of the autophagic process. In any case further research is required to clarify the role of autophagy in the regulation of glioma generation and progression and especially in GICs.

Our work also demonstrates that the pharmacological targeting of the MDK/ALK axis efficiently acts on the population of GICs *in vitro* and in tumor xenografts. Moreover, blockade of MDK/ALK signaling enhances the response of GICs cultures to treatment with TMZ, the benchmark agent for the management of GBM 2, 3, as well as to treatment with THC or a combination of THC:CBD 51. Thus, unlike individual treatments (and specifically blockade of MDK/ALK signaling) that produced a cytostatic effect in GICs cultures, the combined administration of these drugs induced the apoptotic death of GICs leading to an almost complete eradication of this population *in vitro* and to a strikingly enhanced anticancer activity *in vivo*. These observations support the notion that inhibition of the MDK/ALK axis is a useful strategy to target the population of GICs and to improve the response to other antineoplastic agents. Specifically, in this work we tested the activity of two ALK inhibitors (crizotinib and lorlatinib) that are currently used in the clinical practice for the management of other malignancies 34. These findings together with the good safety profile of lorlatinib and especially crizotinib compared with other RTK inhibitors helped to set the bases for a clinical study that is currently evaluating the safety and activity of crizotinib together with temozolomide and radiotherapy in newly-diagnosed glioblastoma (ClinicalTrials.gov Identifier: NCT02270034).

In summary, findings presented in this work show that the MDK/ALK axis plays a key role in the regulation of GICs self-renewal and tumorigenic capacity by controlling the selective autophagic degradation of SOX9. In addition, our data provide strong preclinical evidence that inhibition of this signaling axis may be a potential therapeutic strategy to eliminate this cell population. These findings may provide the rational for the development of novel therapeutic strategies against GBM based on the blockade of the MDK/ALK axis with the aim of targeting the population of GICs.

## Supplementary Material

Supplementary statistical analysis, methods, figures and tables.

## Abbreviations

ALK receptor tyrosine kinase: ALK
Glioma Initiating Cells: GICs
Glioblastoma: GBM
Midkine: MDK
Temozolomide: TMZ
