# Supplementary material for: Midkine signaling maintains the self-renewal and tumorigenic capacity of glioma initiating cells
Source: Theranostics. 2020 Apr 6;10(11):5120–36. doi: 10.7150/thno.41450 (PMC7163450; doi:10.7150/thno.41450)

Supplementary statistical analysis of Figure 5

Crizotinib-treated tumors were significantly different from vehicle-treated tumors from day 10 to day 12 ($P < 0.05$), and from day 13 until the end of the treatment ($P < 0.001$); TMZ-treated tumors were significantly different from vehicle-treated tumors at day 7 ($P < 0.05$), and from day 8 until the end of the treatment ($P < 0.001$); Crizotinib + TMZ-treated tumors were significantly different from vehicle-treated tumors from day 8 until the end of the treatment ($P < 0.001$). Crizotinib + TMZ-treated tumors were significantly different from Crizotinib-treated tumors at day 11 ($P < 0.01$), and from day 12 until the end of the treatment ($P < 0.001$). Crizotinib + TMZ-treated tumors were significantly different from TMZ-treated tumors at day 16 and 17 ($P < 0.05$).

Gene	GH2 Dif.	GH2 GICs	GH11 Dif.	GH11 GICs	12O12 Dif.	12O12 GICs	HCO1 Dif.	HCO1 GICs
SOX2	1,00±0,02	1,05±0,10	1,00±0,16	97,5±8,2 *	1,00±0,19	2,83±0,17 *	1,00±0,24	4,53±1,44 *
BMI1	1,00±0,04	2,22±0,10 *	1,00±0,14	2,06±0,18 *	1,00±0,08	2,74±0,43 *	n.d.	n.d.
MSI1	1,00±0,02	1,18±0,08 *	1,00±0,29	248,5±70,1 *	1,00±0,07	1,72±0,07 *	1,00±0,17	2,16±0,55 *
SURVIVIN	1,00±0,06	4,54±1,00 *	1,00±0,02	2,73±0,03 *	1,00±0,10	4,89±0,57 *	1,00±0,38	17,9±0,48 *
NOTCH3	1,00±0,41	10,65±4,77 *	1,00±0,05	1,90±0,22 *	1,00±0,20	1,79±0,14 *	1,00±0,04	12,7±2,04 *
HES1	1,00±0,07	6,13±1,18 *	1,00±0,07	7,20±0,52 *	1,00±0,09	2,03±0,14 *	1,00±0,26	5,49±0,51 *
NESTIN	1,00±0,05	4,80±1,89 *	1,00±0,12	5,53±1,02 *	1,00±0,35	3,89±0,23 *	1,00±0,16	2,66±0,65 *

Supplementary Table 1. mRNA levels of a panel of stem cell-associated genes (as determined by qPCR) in GICs and their corresponding serum-differentiated counterparts (GH2, GH11, 12O12, HCO1). Data correspond to mRNA levels of each gen and are expressed as the mean fold change from serum-differentiated cells ± SEM. n=3. *$P < 0.05$.

FigS1

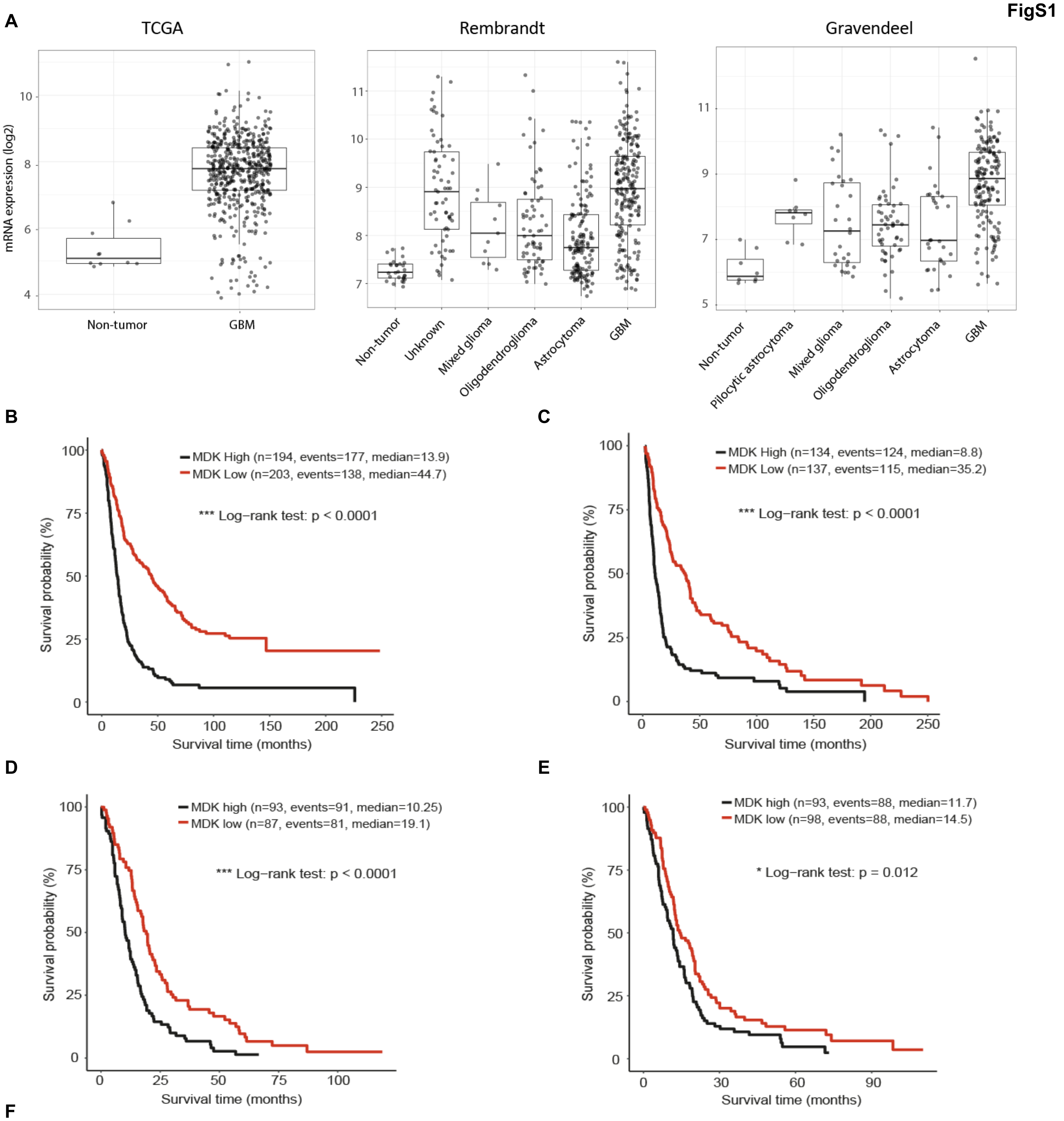

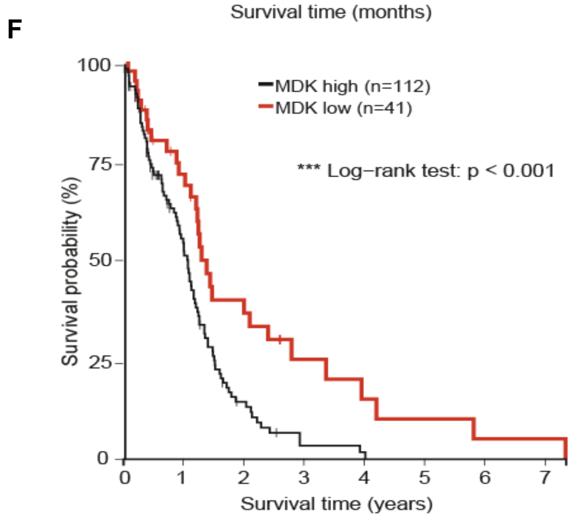

Figure S1: MDK expression and correlation between MDK mRNA levels and survival of human glioma patients in published datasets

A) MDK expression in non-tumor and glioma, including GBM patients, in different data sets from The Cancer Genome Atlas (n=538), Rembrandt (n=537) and Gravendeel (n=284) studies.

B-F) Kaplan-Meier estimator survival analysis separated in two groups, high MDK or low MDK.

B) Survival analysis for glioma Rembrandt study (Low-grade n= 225, GBM n=219)

C) Survival analysis for glioma Gravendeel study (Low-grade n= 117, GBM n=159)

D) Survival analysis for GBM Rembrandt study (n=219)

E) Survival analysis for GBM Lee study (n=191)

F) Survival analysis for glioma Human Protein Atlas portal, Best separation (n= 153)

FigS2

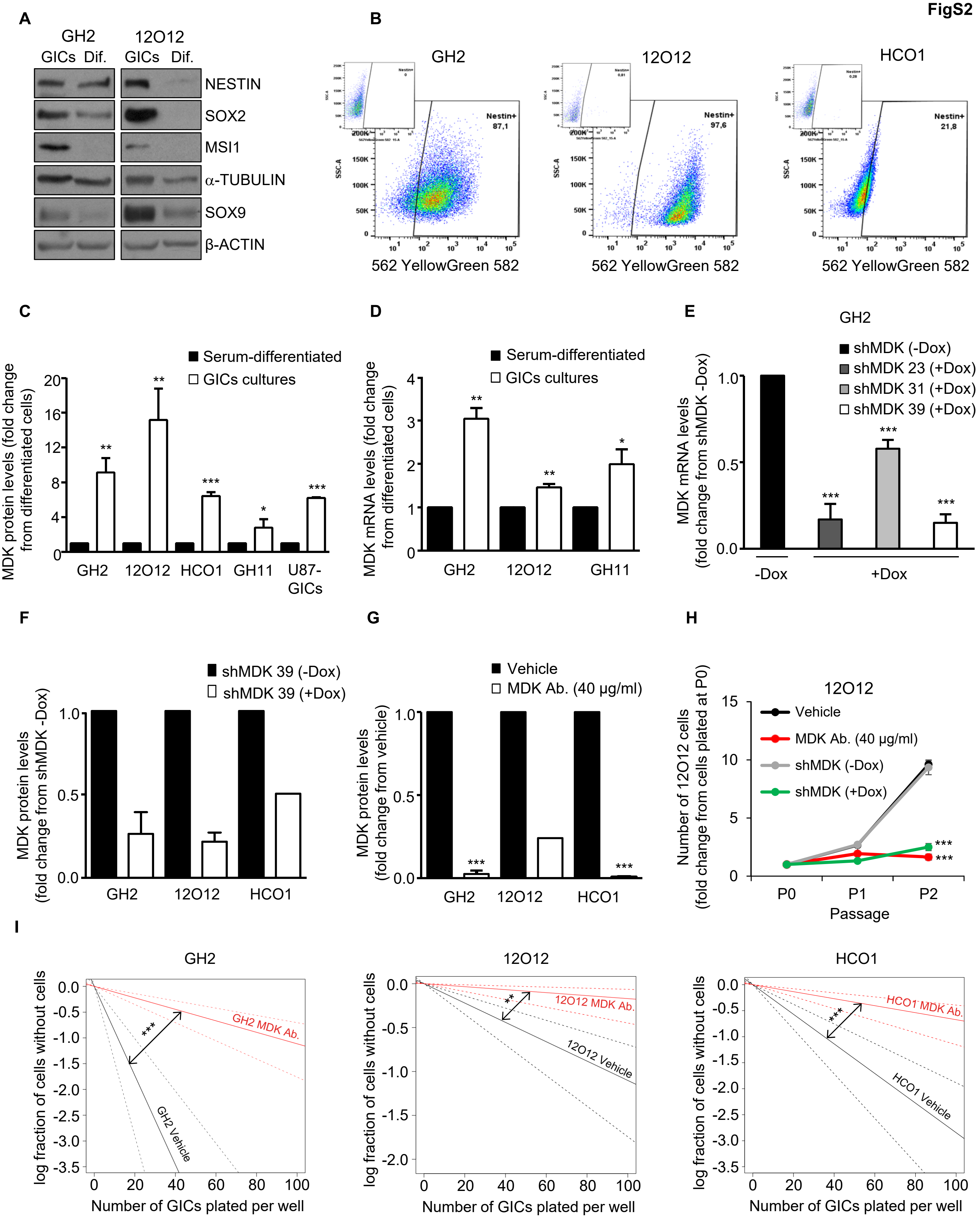

A
GH2
12O12
GICs Dif.
GICs Dif.
NESTIN
SOX2
MSI1
α-TUBULIN
SOX9
β-ACTIN
B
GH2
12O12
HCO1
Nestin+
87,1
97,6
21,8
562 YellowGreen 582
C
Serum-differentiated
GICs cultures
MDK protein levels (fold change from differentiated cells)
GH2
12O12
HCO1
GH11
U87-GICs
D
MDK mRNA levels (fold change from differentiated cells)
E
GH2
shMDK (-Dox)
shMDK 23 (+Dox)
shMDK 31 (+Dox)
shMDK 39 (+Dox)
MDK mRNA levels (fold change from shMDK -Dox)
-Dox
+Dox
F
shMDK 39 (-Dox)
shMDK 39 (+Dox)
MDK protein levels (fold change from shMDK -Dox)
G
Vehicle
MDK Ab. (40 µg/ml)
MDK protein levels (fold change from vehicle)
H
12O12
Vehicle
MDK Ab. (40 µg/ml)
shMDK (-Dox)
shMDK (+Dox)
Number of 12O12 cells (fold change from cells plated at P0)
P0
P1
P2
Passage
I
GH2
12O12
HCO1
log fraction of cells without cells
Number of GICs plated per well
GH2 MDK Ab.
GH2 Vehicle
12O12 MDK Ab.
12O12 Vehicle
HCO1 MDK Ab.
HCO1 Vehicle

Figure S2: MDK inhibition regulates the self-renewal capacity of GICs

(A) NESTIN, SOX2, MUSASHI1 (MSI1) and SOX9 protein levels (as determined by Western blot) of GH2 and 12O12-GICs cultures. A representative Western blot for each cell type is shown (n=2).

(B) Flow cytometry analysis of NESTIN expression in GH2, 12O12 and HCO1-GICs. Representative dot plots of NESTIN immunostaining are shown (the insets correspond to control-unstained cells). Values in the upper right corner of each dot plot correspond to the percentage of NESTIN positive cells in each cell culture.

(C) MDK protein levels (as determined by ELISA) in the medium of GICs cultures or of their corresponding serum-differentiated cells. Data are expressed as the mean fold-change ± SEM (n=3). **P* <0.05; ***P* <0.01; ****P* <0.001 from their corresponding serum-differentiated cells.

(D) MDK mRNA levels (as determined by qPCR) of three different GICs-cultures (GH2, 12O12 and GH11). Data are expressed as the mean fold-change ± SEM (n=3) relative to each serum-differentiated culture. **P* <0.05; ***P* <0.01 from their corresponding serum-differentiated cells.

(E) Effect of MDK silencing (by treating with doxycycline (+Dox.) GH2-GICs stably transduced with three different doxycycline inducible MDK-selective shRNAs, shMDK) on MDK mRNA levels (as determined by qPCR). Data are expressed as the mean fold change ± SEM from the corresponding (-Dox.) cells. (n=3) ****P* < 0.001 from GH2 shMDK (-Dox.) cells.

(F) Effect of expressing shMDK 39 (by treating with doxycycline (+Dox.) cells stably transduced with this doxycycline inducible MDK-selective shRNAs) on MDK protein levels (as determined by ELISA) in the medium of three different GICs-cultures. Data correspond to MDK concentration in the medium and are expressed as the mean fold-change in MDK protein levels relative to the corresponding (-Dox.) shMDK 39 culture ± SEM (n=2).

(G) Effect of incubation with an anti-MDK antibody (MDK Ab., 40 µg/ml, 72 h) on MDK protein levels (as determined by ELISA) in the medium of three different GICs-cultures. Data correspond to MDK protein levels in the medium and are expressed as the mean fold-change relative to vehicle-treated cells. (n=3 GH2 and HCO1). ****P* < 0.001 from GH2 or HCO1 vehicle-treated cells. 12O12 a representative experiment of 2 is shown.

(H) Effect of MDK genetic inhibition (by treating with doxycycline (+Dox.) cells stably-transduced with a doxycycline-inducible MDK-selective shRNA, shMDK) or incubation with an anti-MDK antibody (MDK Ab., 40 µg/ml) during 2 consecutive passages on the growth of 12O12-GICs spheroid cultures. Data correspond to the total number of cells counted upon disaggregation of spheroid cultures in each passage and are expressed as the mean fold-change from the number of cells plated at P0 ± SEM (n = 3). ****P* < 0.001 from vehicle or shMDK (-Dox.) cells.

(I) Effect of incubation with MDK Ab. (40 µg/ml) on the self-renewal ability (as determined by LDA) of GH2, 12O12 and HCO1-GICs. A representative experiment of 2 is showed. ****P* < 0.001 from vehicle-treated cells.

FigS3

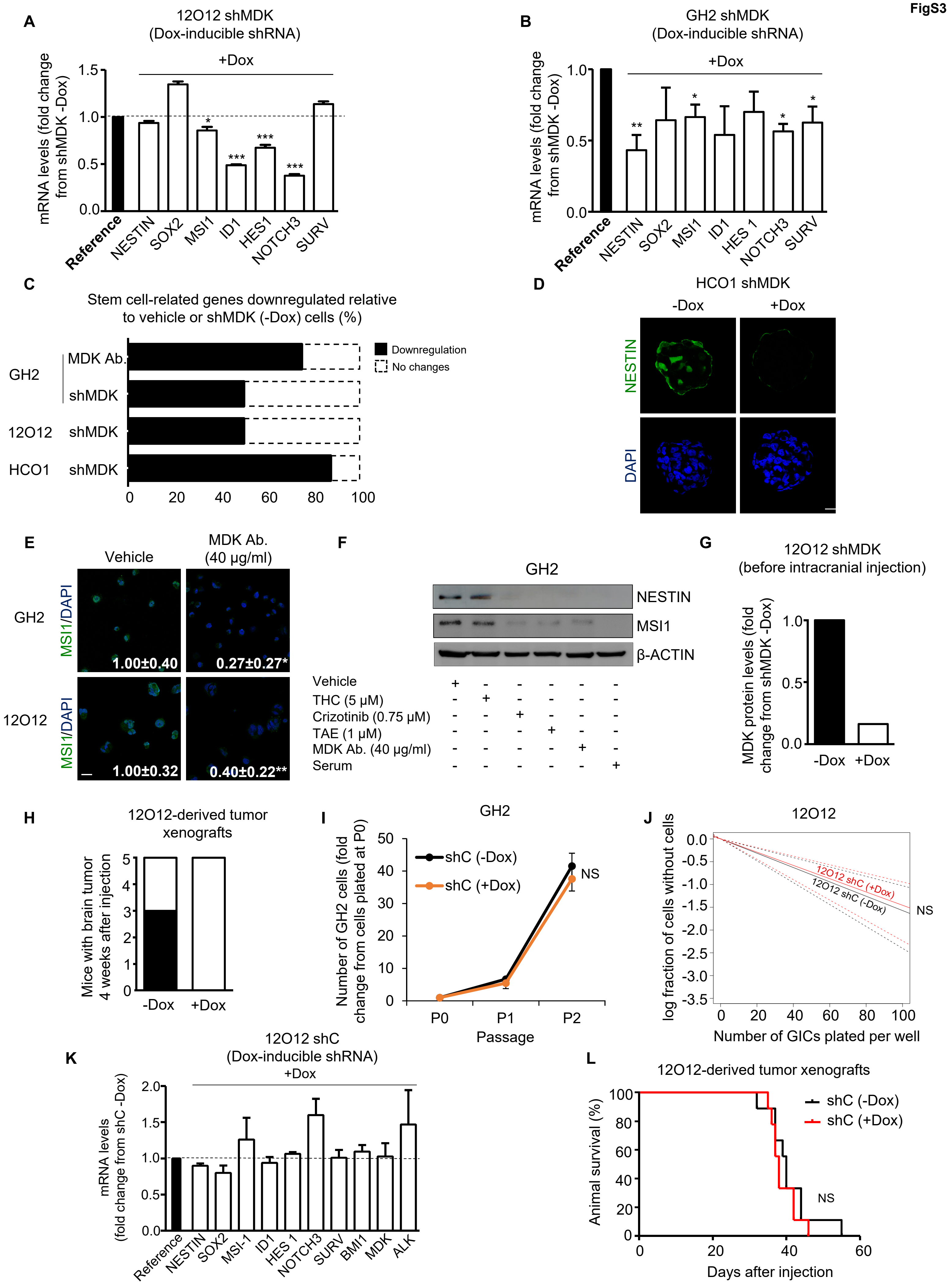
A
12O12 shMDK
(Dox-inducible shRNA)
+Dox
mRNA levels (fold change from shMDK -Dox)
1.5
1.0
0.5
0.0
*
***
***
***
Reference
NESTIN
SOX2
MSI1
ID1
HES1
NOTCH3
SURV
B
GH2 shMDK
(Dox-inducible shRNA)
+Dox
mRNA levels (fold change from shMDK -Dox)
1.0
0.5
0.0
**
*
*
*
Reference
NESTIN
SOX2
MSI1
ID1
HES 1
NOTCH3
SURV
C
Stem cell-related genes downregulated relative to vehicle or shMDK (-Dox) cells (%)
GH2
MDK Ab.
shMDK
12O12
shMDK
HCO1
shMDK
Downregulation
No changes
0
20
40
60
80
100
D
HCO1 shMDK
-Dox
+Dox
NESTIN
DAPI
E
Vehicle
MDK Ab. (40 µg/ml)
GH2
12O12
MSI1/DAPI
1.00±0.40
0.27±0.27*
1.00±0.32
0.40±0.22**
F
GH2
NESTIN
MSI1
β-ACTIN
Vehicle
THC (5 µM)
Crizotinib (0.75 µM)
TAE (1 µM)
MDK Ab. (40 µg/ml)
Serum
G
12O12 shMDK
(before intracranial injection)
MDK protein levels (fold change from shMDK -Dox)
1.0
0.5
0.0
-Dox
+Dox
H
12O12-derived tumor xenografts
Mice with brain tumor 4 weeks after injection
5
4
3
2
1
0
-Dox
+Dox
I
GH2
Number of GH2 cells (fold change from cells plated at P0)
50
40
30
20
10
0
shC (-Dox)
shC (+Dox)
NS
P0
P1
P2
Passage
J
12O12
log fraction of cells without cells
0.0
-0.5
-1.0
-1.5
-2.0
-2.5
-3.0
-3.5
12O12 shC (+Dox)
12O12 shC (-Dox)
NS
0
20
40
60
80
100
Number of GICs plated per well
K
12O12 shC
(Dox-inducible shRNA)
+Dox
mRNA levels (fold change from shC -Dox)
2.0
1.5
1.0
0.5
0.0
Reference
NESTIN
SOX2
MSI-1
ID1
HES 1
NOTCH3
SURV
BMI1
MDK
ALK
L
12O12-derived tumor xenografts
shC (-Dox)
shC (+Dox)
Animal survival (%)
100
80
60
40
20
0
NS
0
20
40
60
Days after injection

Figure S3: Genetic or pharmacological MDK inhibition impairs the self-renewal capacity and tumorigenic properties of GICs

(A-B) Effect of MDK genetic inhibition (by treating with doxycycline (+Dox.) cells stably-transduced with a doxycycline-inducible MDK-selective shRNA, shMDK) on the expression of different stem cell associated genes (as determined by qPCR) of GH2 (A) and 12O12 GICs (B). Data correspond to mRNA levels of each gene and are expressed as the mean fold change from (-Dox.) cells (reference) ± SEM. n=3. *$P < 0.05$; **$P < 0.01$ from GH2 or 12O12 shMDK (-Dox.) cells.

(C) Percentage of stem cell-associated genes downregulated after genetic or pharmacological MDK inhibition in different GICs cultures.

(D) Effect of genetic MDK inhibition (by treating with doxycycline (+Dox.) cells stably-transduced with a doxycycline-inducible shMDK) on NESTIN immunostaining of HCO1 neurospheres. Representative photomicrographs are shown. Scale bar: 20 µm.

(E) Effect of the incubation with MDK Ab. (40 µg/ml, 72 h) on MSI1 expression (as determined by immunofluorescence) of GH2 and 12O12-GICs. Values in each photomicrograph correspond to MSI1-stained area relative to the total number of nuclei (estimated by the DAPI-stained area) in each field and are expressed as mean ± SEM (n=3). Representative photomicrographs are shown. *$P < 0.05$; **$P < 0.01$ from vehicle-treated cells. Scale bar: 20 µm.

(F) Effect of the incubation with MDK Ab. (40 µg/ml), TAE (0.75 µM) and crizotinib (1 µM) for 72 h on the protein levels of NESTIN and MSI1. n=2. A representative Western blot is shown.

(G) Effect of MDK genetic inhibition [by inducing the expression of doxycycline-inducible shMDK (+Dox.)] on MDK protein levels (as determined by ELISA) presented in the medium of shMDK 39 stably-transduced 12O12 GICs 24 hours before their intracranial injection into the striatum of nude mice.

(H) Effect of MDK genetic inhibition [by inducing the expression of doxycycline-inducible shMDK (+Dox.)] on the percentage of nude mice with detectable tumors by MRI (black bars) 4 weeks after intracranial injection of 7.5×10^4 12O12 shMDK-GICs (n=6).

(I-K) Effect of doxycycline (+Dox.) on the growth (I), the self-renewal capacity (as determined by LDA experiments, J) and the mRNA levels of a panel of stem cell markers as well as of *MDK* and *ALK* (K) of 12O12-GICs stably-transduced with a doxycycline inducible control-shRNA (shC). NS: statistically non-significant differences.

(L) Effect of the treatment with doxycycline (+Dox.) and of the subsequent administration of a doxycycline-enriched diet, on the survival of mice bearing intracranial tumors generated by the injection of 7.5×10^4 12O12 stably transduced with a doxycycline inducible control-shRNA (shC). (n=9). NS: non-significant statistical differences.

FigS4

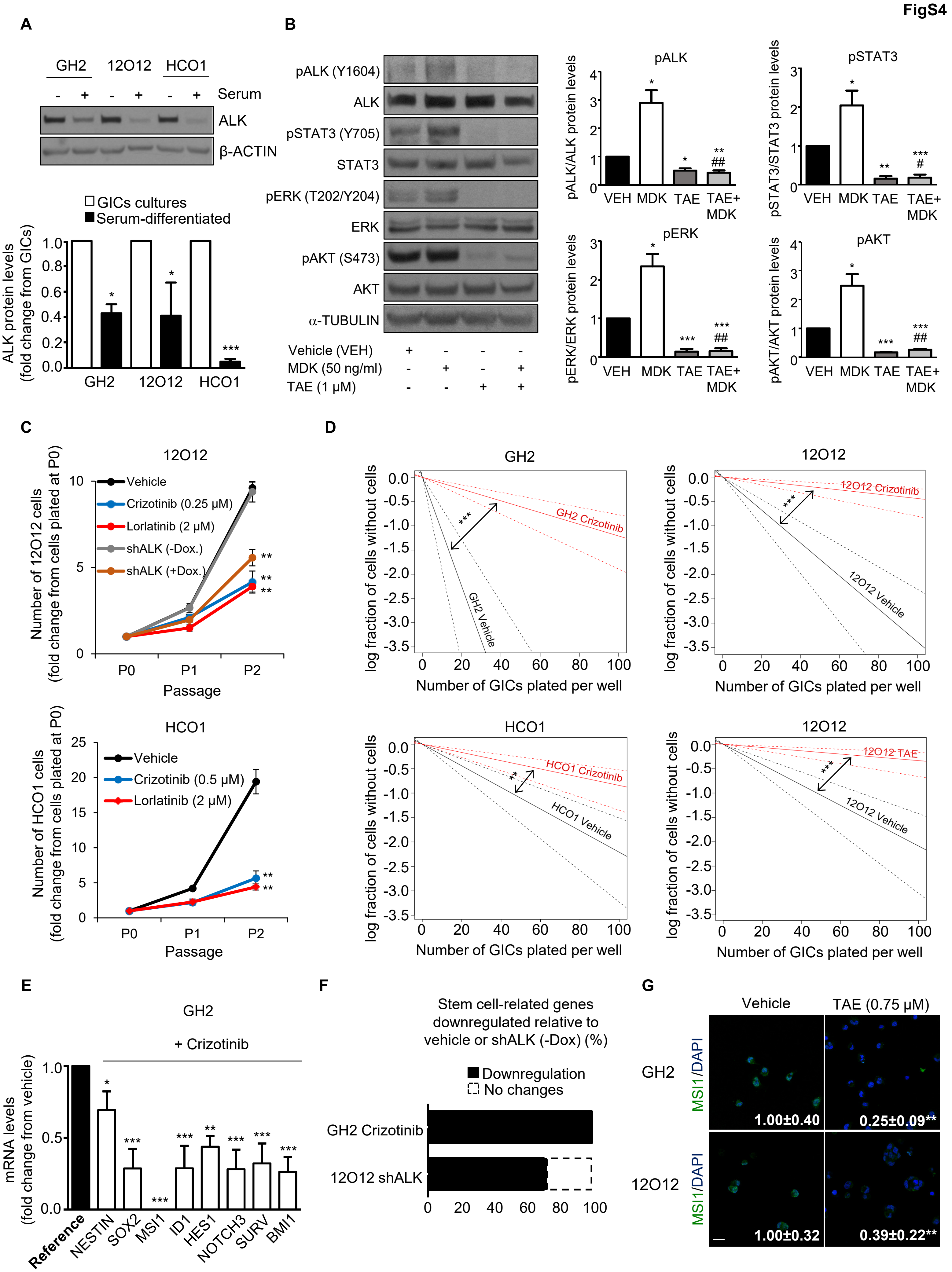
A
GH2
12O12
HCO1
Serum
ALK
β-ACTIN
GICs cultures
Serum-differentiated
ALK protein levels (fold change from GICs)
B
pALK (Y1604)
ALK
pSTAT3 (Y705)
STAT3
pERK (T202/Y204)
ERK
pAKT (S473)
AKT
α-TUBULIN
Vehicle (VEH)
MDK (50 ng/ml)
TAE (1 μM)
pALK
pALK/ALK protein levels
pSTAT3
pSTAT3/STAT3 protein levels
pERK
pERK/ERK protein levels
pAKT
pAKT/AKT protein levels
VEH
MDK
TAE
TAE+ MDK
C
12O12
Vehicle
Crizotinib (0.25 μM)
Lorlatinib (2 μM)
shALK (-Dox.)
shALK (+Dox.)
Number of 12O12 cells (fold change from cells plated at P0)
Passage
HCO1
Crizotinib (0.5 μM)
Number of HCO1 cells (fold change from cells plated at P0)
D
GH2 Crizotinib
GH2 Vehicle
12O12 Crizotinib
12O12 Vehicle
HCO1 Crizotinib
HCO1 Vehicle
12O12 TAE
log fraction of cells without cells
Number of GICs plated per well
E
GH2
+ Crizotinib
mRNA levels (fold change from vehicle)
Reference
NESTIN
SOX2
MSI1
ID1
HES1
NOTCH3
SURV
BMI1
F
Stem cell-related genes downregulated relative to vehicle or shALK (-Dox) (%)
Downregulation
No changes
GH2 Crizotinib
12O12 shALK
G
Vehicle
TAE (0.75 μM)
MSI1/DAPI
1.00±0.40
0.25±0.09**
1.00±0.32
0.39±0.22**

Figure S4: MDK effects on GIC rely on ALK

(A) ALK protein levels in different GICs cultures and their corresponding serum-differentiated-cells as determined by Western blot. A representative experiment of three (upper panel) with the corresponding densitometric quantification (bottom panel) is shown. **P* < 0.05; ****P* < 0.001 from GICs cultures.

(B) Effect of TAE (1 µM, 30 min) and exogenous MDK (MDK, 50 ng/ml, 30 min) on ALK, STAT3, AKT and ERK phosphorylation of GH2-GICs. A representative Western blot (left panel) with the corresponding densitometric quantification (right panel) is shown (n=3). Data are expressed as the phosphorylated/total protein ratio for each condition relative to vehicle-treated GH2-GICs (n=3). **P* < 0.05; ***P* < 0.01; ****P* < 0.001 from vehicle-treated cells. $^{\#}$ *P* < 0.05; $^{\#\#}$ *P* < 0.01 from MDK-treated cells.

(C) Effect of ALK genetic inhibition (by treating with doxycycline (+Dox.) cells stably-transduced with a doxycycline-inducible ALK-selective shRNA; shALK) or pharmacological ALK inhibition (by incubation with the ALK tyrosine-kinase inhibitors crizotinib and lorlatinib) during 2 consecutive passages on the growth of 12O12 (upper panel) and HCO1 (bottom panel)-GICs. Data correspond to the total number of cells counted upon disaggregation of spheroid cultures in each passage and are expressed as the mean fold-change from the number of cells plated at P0 ± SEM. n = 3. ***P* < 0.01 from vehicle or shALK (-Dox.) cells.

(D) Effect of crizotinib (0.5 µM) and TAE (0.75 µM) on the self-renewal ability (as determined by LDA) of GH2, 12O12 and HCO1-GICs. In each case a representative experiment of 2 is shown. ***P* < 0.01 ****P* < 0.001 from vehicle-treated cells.

(E) Effect of TAE (0.75 µM, 72 h) on MSI1 expression (as determined by immunofluorescence) of GH2 and 12O12-GICs. Values in each photomicrograph correspond to MSI1-stained area relative to the total number of nuclei (estimated by the DAPI-stained area) in each field and are expressed as mean ± SEM. Representative photomicrographs are shown. ***P* < 0.01 from vehicle-treated cells. Scale bar: 20 µm.

(F) Effect of crizotinib (0.5 µM, 72 h) on the expression of different stem cell-related genes (as determined by qPCR) of GH2-GICs. Data correspond to mRNA levels of each gene and are expressed as the mean fold change from vehicle-treated cells (reference) ± SEM. n=3. **P* < 0.05; ***P* < 0.01 and ****P* < 0.001 from vehicle-treated cells.

(G) Percentage of stem cell-associated genes downregulated after genetic (12O12-GICs) or pharmacological (GH2-GICs) ALK inhibition in GICs cultures.

FigS5

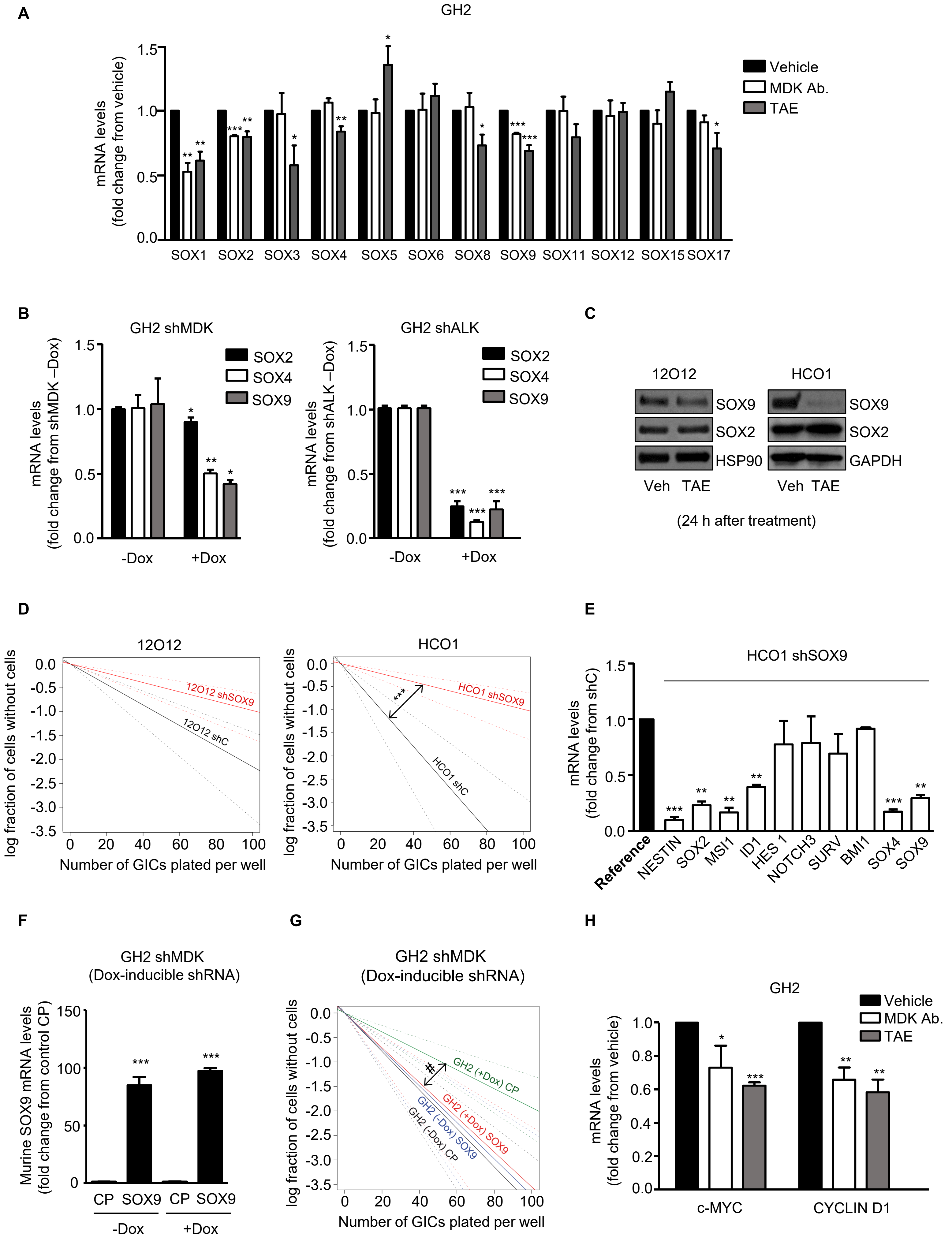
A
GH2
mRNA levels
(fold change from vehicle)
Vehicle
MDK Ab.
TAE
SOX1
SOX2
SOX3
SOX4
SOX5
SOX6
SOX8
SOX9
SOX11
SOX12
SOX15
SOX17
B
GH2 shMDK
mRNA levels
(fold change from shMDK –Dox)
SOX2
SOX4
SOX9
-Dox
+Dox
GH2 shALK
mRNA levels
(fold change from shALK –Dox)
C
12O12
HCO1
SOX9
SOX2
HSP90
GAPDH
Veh TAE
(24 h after treatment)
D
12O12
log fraction of cells without cells
Number of GICs plated per well
12O12 shSOX9
12O12 shC
HCO1
HCO1 shSOX9
HCO1 shC
E
HCO1 shSOX9
mRNA levels
(fold change from shC)
Reference
NESTIN
SOX2
MSI1
ID1
HES 1
NOTCH3
SURV
BMI1
SOX4
SOX9
F
GH2 shMDK
(Dox-inducible shRNA)
Murine SOX9 mRNA levels
(fold change from control CP)
CP
SOX9
-Dox
+Dox
G
GH2 shMDK
(Dox-inducible shRNA)
GH2 (+Dox) CP
GH2 (+Dox) SOX9
GH2 (-Dox) SOX9
GH2 (-Dox) CP
H
GH2
mRNA levels
(fold change from vehicle)
c-MYC
CYCLIN D1

Figure S5. MDK/ALK signaling axis regulates the maintenance of the stem-like properties of GICs by controlling SOX9 protein levels

(A) Effect of the incubation with MDK Ab. (40 µg/ml) or TAE (0.75 µM) for 72 h on mRNA levels (as determined by qPCR) of different SOX family members of GH2-GICs. Data are expressed as the mean fold change from vehicle-treated cells (n=3) ± SEM. *P <0.05; **P <0.01; ***P <0.001 from vehicle-treated cells.

(B) Effect of treatment with doxycycline (+Dox.) during two consecutive passages on SOX2, SOX4 and SOX9 mRNA levels (as determined by real-time quantitative PCR) of GH2-GICs stably-transduced with a doxycycline-inducible MDK-selective (shMDK, left panel) or ALK-selective (shALK, right panel) shRNA. Data are expressed as the mean fold change from the corresponding (-Dox.) cells ± SEM (n=3). *P <0.05; **P <0.01 from GH2 shMDK (-Dox.) cells and ***P <0.001 from GH2 shALK (-Dox.) cells.

(C) Effect of the incubation with TAE (0.75 µM) for 24 h on SOX2 and SOX9 protein levels of 12O12 and HCO1-GICs. A representative Western blot is shown. (n=2)

(D) Effect of nucleofection with a pLKO plasmid encoding a control (shC) or SOX9-selective (shSOX9) shRNA on the self-renewal ability (as determined by LDA experiments) of 12O12-GICs (left panel) or HCO1-GICs (right panel). ***P < 0.001 from pLKO shC-transduced cells (72 h).

(E) Effect of nucleofection with shC or shSOX9 pLKO plasmid (72 h) on mRNA levels (as determined by qPCR) of a panel of stem cell-associated genes in HCO1-GICs. Data correspond to mRNA levels of each gen and are expressed as the mean fold change from shC-transduced cells (reference) ± SEM. n=3. *P < 0.05; **P < 0.01; **P < 0.001 from shC-transduced cells.

(F) Effect of nucleofection with a control plasmid (pWPXL GFP, control) or a plasmid encoding murine SOX9 (pWPXL SOX9 plasmid, SOX9) on murine SOX9 mRNA levels (as determined by qPCR) of GH2-shC or GH2-shMDK-GICs (n=3).

(G) Effect of MDK genetic inhibition (by treating with doxycycline cells stably-transduced with a doxycycline-inducible MDK-selective shRNA, shMDK) and nucleofection with a control plasmid (CP) or a plasmid encoding murine SOX9 (SOX9) on the self-renewal ability (as determined by LDA) of GH2-GICs. n=2; a representative experiment is shown. $^{\#}P$ < 0.05 from shMDK (+Dox) CP cells. Other symbols of significance are omitted for clarity. Full χ^2 statistical analysis is included in LDA statistics supplementary section.

(H) Effect of the incubation with an anti-MDK antibody (MDK Ab.; 40 µg/ml) and TAE (0.75 µM) for 72h on *cMYC* and *CYCLIND1* mRNA levels (as determined by qPCR) of GH2-GICs. Data are expressed as the mean fold change from vehicle-treated cells ± SEM (n=3). *P < 0.05; **P < 0.01; ***P < 0.001 from vehicle-treated cells.

FigS6

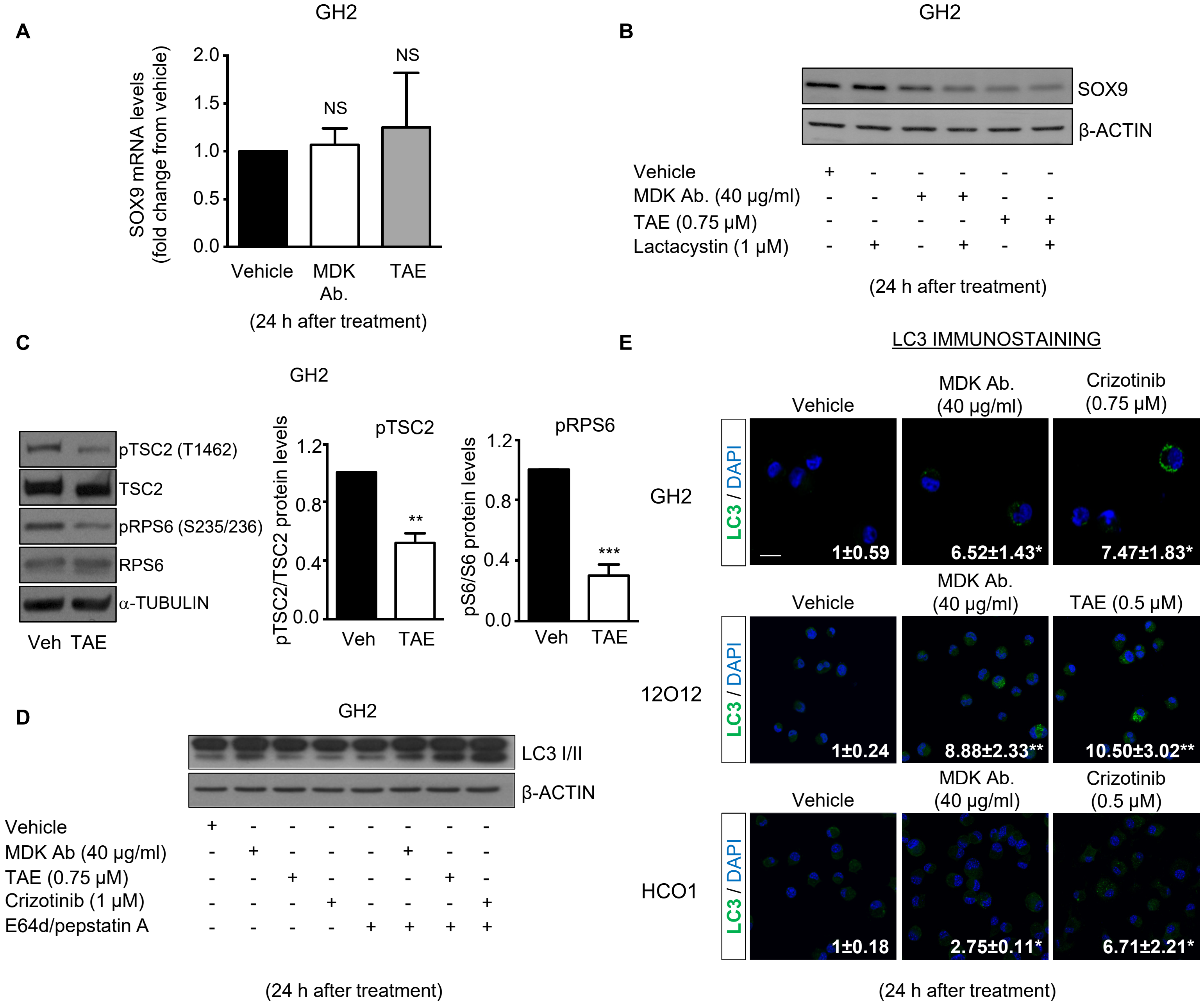

Figure S6: Blockade of the MDK/ALK axis in GICs triggers SOX9 degradation via autophagy stimulation
(A) Effect of the incubation with an anti-MDK antibody (MDK Ab; 40 µg/ml) and TAE (0.75 µM) for 24h on SOX9 mRNA levels (as determined by qPCR) of GH2-GICs. Data are expressed as the mean fold change from vehicle-treated cells ± SEM (n=3). NS: statistically non-significant differences.
(B) Effect of the incubation with MDK Ab (40 µg/ml) or TAE (0.75 µM) for 24 h on SOX9 protein levels of GH2 GICs cultures untreated or pretreated with lactacystin (1 µM) for 1 h (n=3).
(C) Effect of the incubation with TAE (0.75 µM) for 30 minutes on the phosphorylation levels of TSC2 and S6 proteins on GH2-GICs. A representative experiment is shown (n=3; left panel). Right panel: Data correspond to the densitometric analysis of the levels of each phosphorylated protein relative to total levels of that protein and are expressed as the mean fold change ± SEM relative to vehicle treated cells (right panel). $^{**}P < 0.01$; $^{***}P < 0.001$ from vehicle-treated cells.
(D) Effect of the incubation with MDK Ab. (40 µg/ml), TAE (0.75 µM) and crizotinib (1 µM) for 24 hours on LC3 lipidation in the presence of E64d (10 mM) and pepstatin A (10 mg/ml) of GH2 GICs-enriched-cultures. A representative Western blot from n=3 is shown.
(E) Effect of the incubation with MDK Ab. (40 µg/ml), TAE (0.5 µM) and Crizotinib (0.5 µM) for 24 hours on LC3 immunostaining of GH2, 12O12 and HCO1-GICs. Values in the bottom right corner at each photomicrograph correspond to the ratio between the sum of the LC3 vesicle areas (ΣA_{ves}) and the total cell area (A_{cell}) calculated per each cell. Representative images of each experimental condition are shown (n=3). $^{*}P < 0.05$; $^{**}P < 0.01$ from vehicle-treated cells.

FigS7

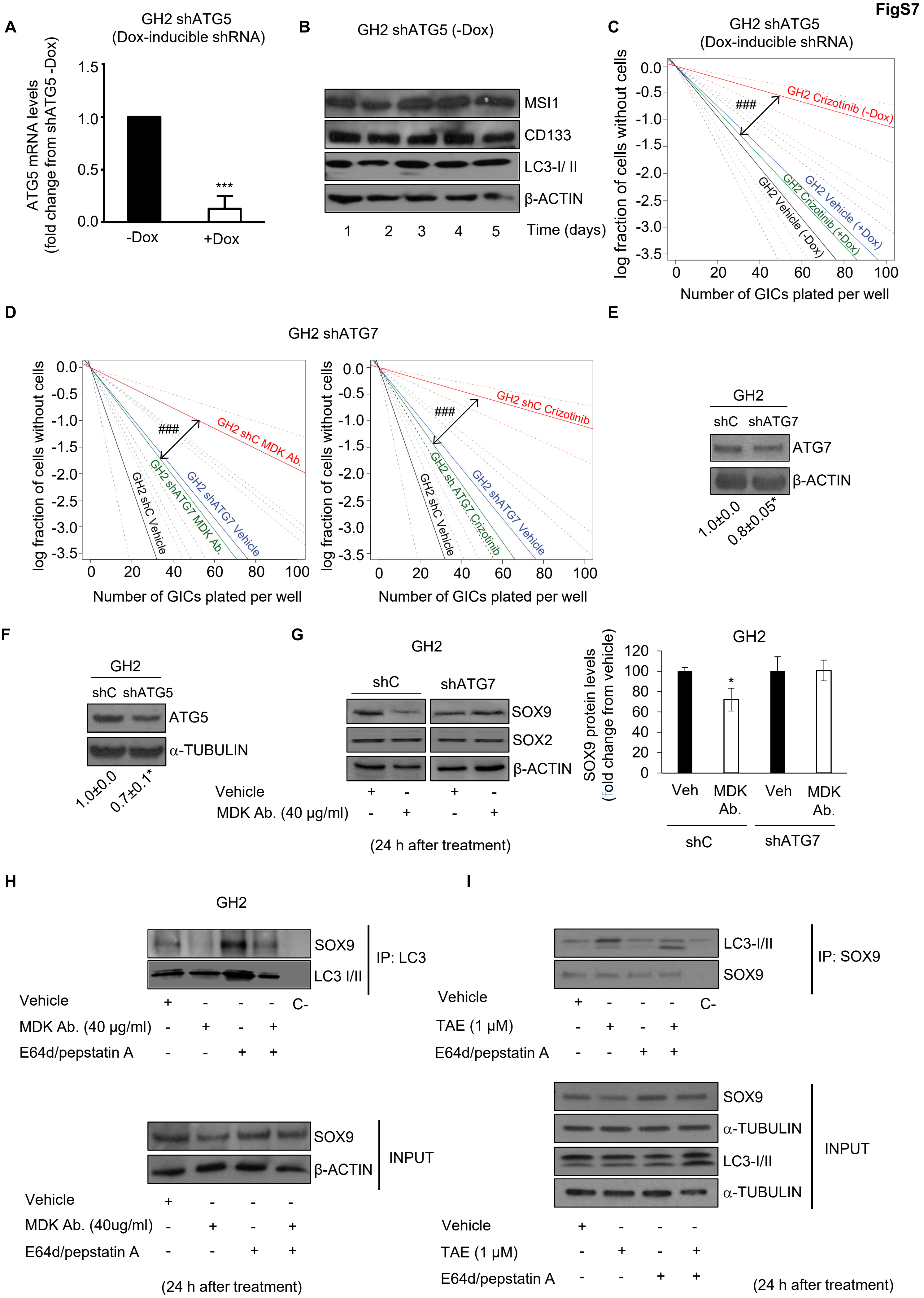
A
GH2 shATG5
(Dox-inducible shRNA)
ATG5 mRNA levels
(fold change from shATG5 -Dox)
1.5
1.0
0.5
0.0
***
-Dox
+Dox
B
GH2 shATG5 (-Dox)
MSI1
CD133
LC3-I/ II
β-ACTIN
1
2
3
4
5
Time (days)
C
GH2 shATG5
(Dox-inducible shRNA)
log fraction of cells without cells
0.0
-0.5
-1.0
-1.5
-2.0
-2.5
-3.0
-3.5
###
GH2 Crizotinib (-Dox)
GH2 Vehicle (+Dox)
GH2 Crizotinib (+Dox)
GH2 Vehicle (-Dox)
0
20
40
60
80
100
Number of GICs plated per well
D
GH2 shATG7
log fraction of cells without cells
###
GH2 shC MDK Ab.
GH2 shATG7 Vehicle
GH2 shATG7 MDK Ab.
GH2 shC Vehicle
Number of GICs plated per well
log fraction of cells without cells
###
GH2 shC Crizotinib
GH2 shATG7 Vehicle
GH2 sh ATG7 Crizotinib
GH2 shC Vehicle
Number of GICs plated per well
E
GH2
shC
shATG7
ATG7
β-ACTIN
1.0±0.0
0.8±0.05*
F
GH2
shC
shATG5
ATG5
α-TUBULIN
1.0±0.0
0.7±0.1*
G
GH2
shC
shATG7
SOX9
SOX2
β-ACTIN
Vehicle + - + -
MDK Ab. (40 µg/ml) - + - +
(24 h after treatment)
GH2
SOX9 protein levels
(fold change from vehicle)
120
100
80
60
40
20
0
*
Veh
MDK Ab.
Veh
MDK Ab.
shC
shATG7
H
GH2
SOX9
LC3 I/II
IP: LC3
Vehicle + - - - C-
MDK Ab. (40 µg/ml) - + - +
E64d/pepstatin A - - + +
SOX9
β-ACTIN
INPUT
Vehicle + - - -
MDK Ab. (40ug/ml) - + - +
E64d/pepstatin A - - + +
(24 h after treatment)
I
LC3-I/II
SOX9
IP: SOX9
Vehicle + - - - C-
TAE (1 µM) - + - +
E64d/pepstatin A - - + +
SOX9
α-TUBULIN
LC3-I/II
α-TUBULIN
INPUT
Vehicle + - - -
TAE (1 µM) - + - +
E64d/pepstatin A - - + +
(24 h after treatment)

Figure S7: Autophagy stimulation is necessary for the loss of the stem-like properties of GICs induced by MDK/ALK axis inhibition

(A) Effect of ATG5 genetic inhibition (by treating with doxycycline (+Dox.) cells stably-transduced with doxycycline-inducible ATG5-selective shRNA, shATG5) on ATG5 mRNA levels (as determined by qPCR) of GH2-GICs. Data are expressed as the mean fold change from the corresponding doxycycline non-treated cells ± SEM. n=3 ***P* < 0.01 from GH2 shATG5 (-Dox.) cells.

(B) MUSASHI-1 (MSI1), CD133 and LC3-I/II expression as determined by Western blot of GH2 shATG5 (-Dox.)-treated cells at different time points.

(C) Effect of the incubation with crizotinib (0.75 µM) and of genetic inhibition of autophagy (by treating with doxycycline (+Dox.) cells stably-transduced with doxycycline-inducible ATG5-selective shRNA, shATG5) on the self-renewal ability (as determined by LDA) of GH2-GICs. A representative experiment from n=2 is shown. ###*P* < 0.001 from Crizotinib-treated (-Dox) cells. Other symbols of significance are omitted for clarity. Full χ^2 statistical analysis is included in LDA statistics supplementary section.

(D) Effect of the incubation with an anti-MDK antibody (MDK Ab., 40 µg/ml, left panel) and crizotinib (0.75 µM, right panel) on the self-renewal ability (as determined by LDA) of GH2-GICs stably transduced with a control (shC) or a constitutive ATG7-selective (shATG7) shRNA. A representative experiment from n=2 is showed. ###*P* < 0.001 from MDK Ab-treated shC cells and ###*P* < 0.001 from Crizotinib-treated shC cells. Other symbols of significance are omitted for clarity. Full χ^2 statistical analysis is included in LDA statistics supplementary section.

(E) ATG7 protein levels (as determined by Western blot) on GH2-GICs stably transduced with a control (shC) or a constitutive ATG7-selective (shATG5) shRNA. A representative western blot is shown. Values correspond to the densitometric analysis of ATG7/α-TUBULIN ratio and are expressed as the mean fold change ± SEM from shC-transduced cells. **P* < 0.05.

(F) ATG5 protein levels (as determined by Western blot) on GH2-GICs stably transduced with a control (shC) or a constitutive ATG5-selective (shATG5) shRNA. A representative western blot is shown. Values correspond to the densitometric analysis of ATG5/α-TUBULIN ratio and are expressed as the man fold change ± SEM from shC-transduced cells. **P* < 0.05.

(G) Effect of the incubation with an anti-MDK antibody (MDK Ab., 40 µg/ml) on the protein levels (as determined by Western blot) of SOX9, SOX2, SOX4 in GH2-GICs stably transduced with a control (shC) or a constitutive ATG7-selective (shATG7) shRNA. Left panel**:** A representative Western blot experiment from n=3 is showed. Right panel: Densitometric analysis of SOX9 protein levels. Data are expressed as SOX9/β-ACTIN ratio and are represented as the mean fold change ± SEM from GH2 shC vehicle-treated cells. **P* < 0.05 from vehicle-treated cells.

(H) SOX9 protein levels after immunoprecipitation of GH2-GICs cultures untreated or pretreated for 1 h with E64d/pepstatin A (PA) and subsequently incubated with an anti-MDK antibody (40 µg/ml) for 24 h (n=2).

(I) LC3-I/II protein levels after immunoprecipitation of GH2-GICs cultures untreated or pretreated for 1 h with E64d/pepstatin A (PA) and subsequently incubated with TAE (1 µM) for 24 h (n=4).

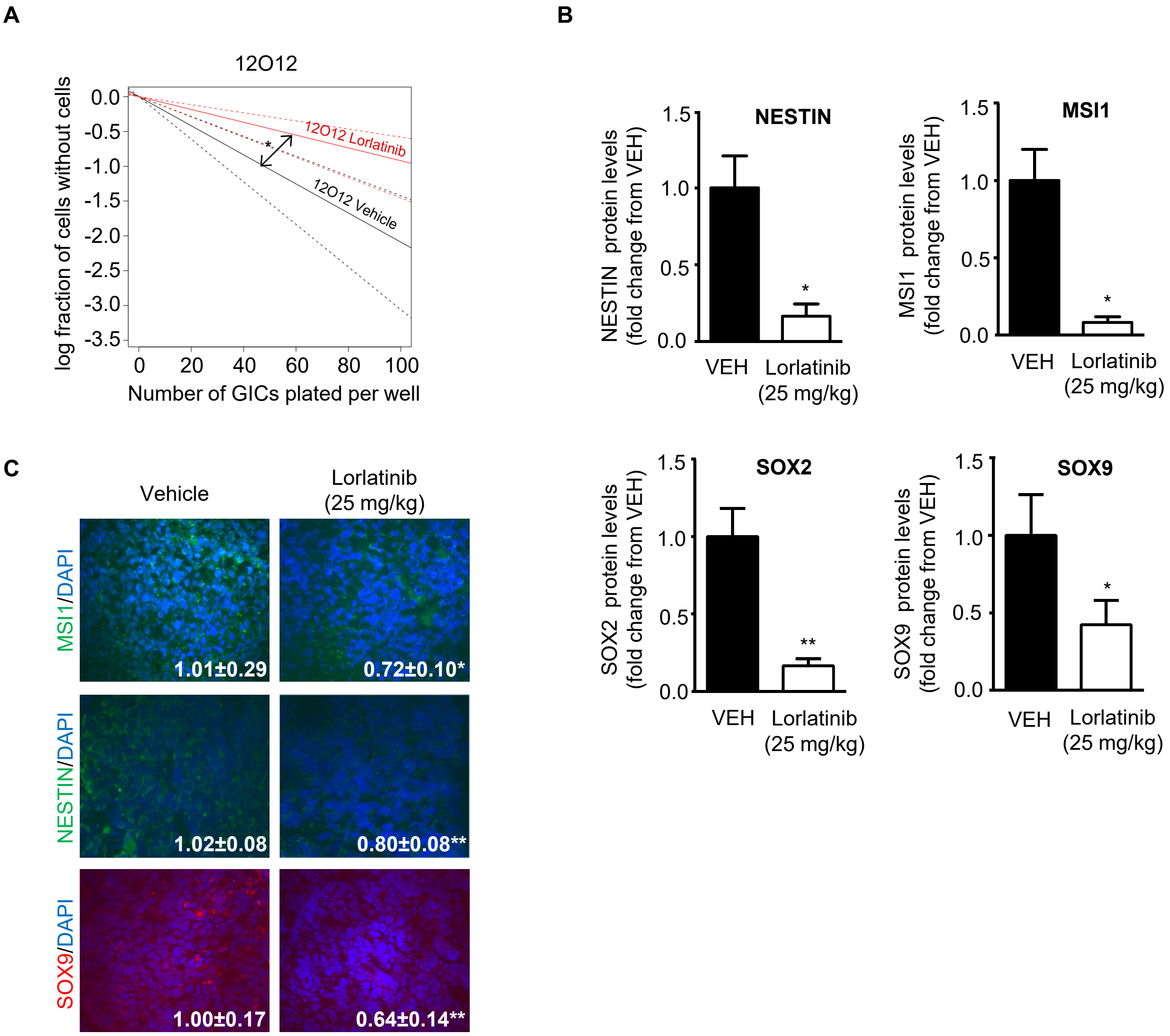

Figure S8: ALK pharmacological inhibition targets the population of GICs *in vivo*

(A) Effect of lorlatinib (1 µM) on the self-renewal ability (as determined by limiting dilution assays, LDA) of 12O12-GICs. *P < 0.05 from vehicle-treated cells.

(B) Effect of lorlatinib (25 mg/kg daily oral administration) on the expression (as determined by Western blot) of NESTIN, MUSASHI-1 (MSI1), SOX2 and SOX9 in samples derived from tumor xenografts generated by the subcutaneous injection of 2 x 10^6 12O12-GICs. Data correspond to the densitometric analysis of the corresponding protein/β-ACTIN ratio relative to one of the vehicle-treated samples and are expressed as the mean fold change ± SEM relative to vehicle treated tumors. n=6 (vehicle, VEH) and n=5 (lorlatinib)-treated tumors. * P < 0.05 and ** P < 0.01 from vehicle-treated tumors.

(C) Effect of lorlatinib (25 mg/kg daily oral administration) on MSI1 (upper panel), NESTIN (middle panel) and SOX9 (lower panel) immunostaining of 12O12 GICs-derived subcutaneous tumor xenografts. Values in the bottom right corner at each photomicrograh correspond to MSI, NESTIN or SOX9-stained area relative to the number of nuclei in each field (estimated by the DAPI-stained area) and are expressed as mean ± SEM (10 photomicrographs/fields per tumor of 4 different tumors for each experimental condition were analyzed). * P < 0.05; ** P < 0.01 from vehicle-treated tumors.

FigS9

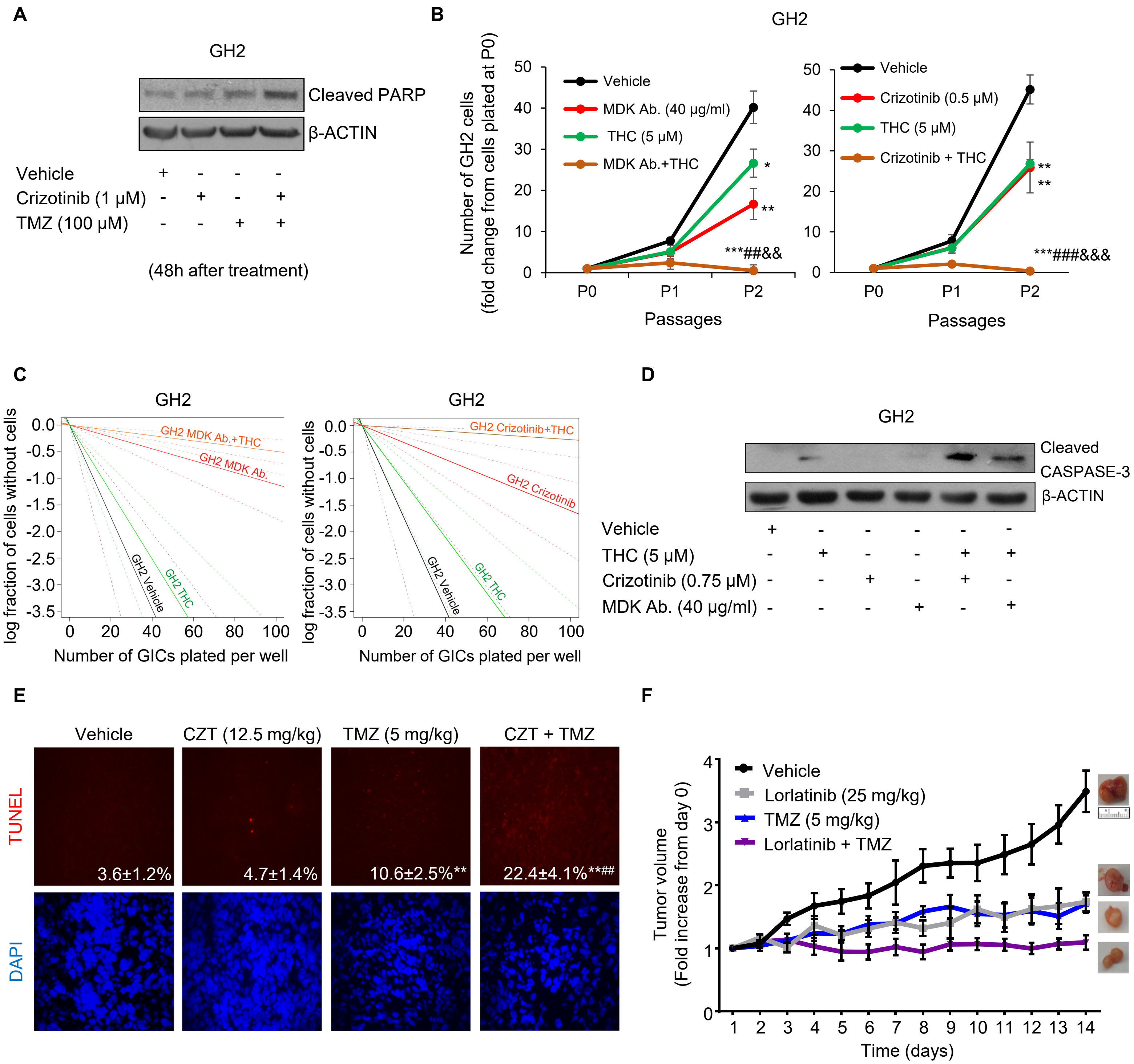
A
GH2
Cleaved PARP
β-ACTIN
Vehicle + - - -
Crizotinib (1 μM) - + - +
TMZ (100 μM) - - + +
(48h after treatment)
B
GH2
Number of GH2 cells (fold change from cells plated at P0)
Vehicle
MDK Ab. (40 μg/ml)
THC (5 μM)
MDK Ab.+THC
***##&&
Crizotinib (0.5 μM)
Crizotinib + THC
***###&&&
Passages
C
GH2
log fraction of cells without cells
GH2 MDK Ab.+THC
GH2 MDK Ab.
GH2 Vehicle
GH2 THC
GH2 Crizotinib+THC
GH2 Crizotinib
Number of GICs plated per well
D
GH2
Cleaved CASPASE-3
β-ACTIN
Vehicle + - - - - -
THC (5 μM) - + - - + +
Crizotinib (0.75 μM) - - + - + -
MDK Ab. (40 μg/ml) - - - + - +
E
Vehicle
CZT (12.5 mg/kg)
TMZ (5 mg/kg)
CZT + TMZ
TUNEL
DAPI
3.6±1.2%
4.7±1.4%
10.6±2.5%**
22.4±4.1%**##
F
Tumor volume (Fold increase from day 0)
Vehicle
Lorlatinib (25 mg/kg)
TMZ (5 mg/kg)
Lorlatinib + TMZ
Time (days)

Figure S9: Blockade of the MDK/ALK axis enhances the anticancer activity of cannabinoids and TMZ in GICs-derived tumors.

(A) Effect of the treatment with Crizotinib (1 µM) and TMZ (100 µM) on apoptosis (as determined by the cleavage of the CASPASE 3 substrate PARP) of GH2-GICs (48h). n=3.

(B) Effect of the treatment with THC and MDK Ab (left panel) or Crizotinib (right panel) during 2 consecutive passages on the growth of 12O12-GICs. Data are expressed as the mean fold-change from the number of cells plated at P0 ± SEM. n=3. $^{*}P < 0.05$, $^{**}P < 0.01$ and $^{***}P <0.001$ from vehicle-treated cells; $^{\#\#}P < 0.01$ or $^{\#\#\#}P < 0.001$ from THC-treated cells; $^{\&\&}P <0.01$ or $^{\&\&\&}P <0.001$ from anti-MDK antibody-treated cells (left panel) or Crizotinib-treated cells (right panel).

(C) Effect of the treatment with THC (5 µM) and MDK Ab. (40 µg/ml, left panel) or Crizotinib (0.5 µM, right panel) on the self-renewal ability (as determined by LDA) of GH2-GICs. A representative experiment of 2 is shown. Symbols of significance are omitted for clarity. Symbols of significance are omitted for clarity. Full χ^2 statistical analysis is included in LDA statistics supplementary section.

(D) Effect of the treatment with THC (5 µM), MDK Ab. (40 µg/ml) and Crizotinib (0.75 µM) on apoptosis (as determined by the cleavage of the CASPASE 3 substrate PARP) of GH2-GICs (5 d). n=3.

(E) Effect of crizotinib (CZT, 12.5 mg/kg daily oral administration)and TMZ (5 mg/kg twice a week IP administration) on the number of apoptotic cells (as determined by TUNEL) in samples derived from tumor xenografts generated by the subcutaneous injection of 2 x 10^6 12O12-GICs in the flank of nude mice. Representative images of tumors of each experimental condition are shown. Values in the bottom right corner at each photomicrograph correspond to the number of TUNEL-positive nuclei relative to the total number of nuclei per field determined by DAPI staining and are expressed as the mean ± SEM (6 photomicrographs/fields per tumor of 5 different tumors for each experimental condition were analyzed). ** $P < 0.01$ from vehicle-treated tumors. $^{\#\#}$ $P < 0.01$ from crizotinib-treated tumors.

(F) Effect of treatment with lorlatinib (25 mg/kg daily oral administration), TMZ (5 mg/kg twice a week IP administration) on the growth of glioma xenografts generated by subcutaneous injection of 2 x 10^6 12O12 GICs in the flank of nude mice (mean ± SEM; n=5-6 mice for each condition). Representative pictures of the tumor xenografts in the last day of the treatment are shown for each experimental condition. Symbols of significance are omitted for clarity. Lorlatinib-treated tumors were significantly different from vehicle-treated tumors from day 8 to day 10 ($P < 0.01$), and from day 11 until the end of the treatment ($P < 0.001$); TMZ-treated tumors were significantly different from vehicle-treated tumors from day 8 to day 10 ($P < 0.05$), and from day 11 until the end of the treatment ($P < 0.001$); lorlatinib + TMZ-treated tumors were significantly different from vehicle-treated tumors at day 5 and day 6 ($P < 0.05$) and from day 7 until the end of the treatment ($P < 0.001$).

Supplementary methods information

Gene	Forward primer (5’-3’)	Reverse primer (5’-3’)
ALK	CAAGTCCTTCCTCCGAGAGAC	AGCCACGTGCAGAAGGTC
ATG5	CAACTTGTTTCACGCTATATCAGG	CACTTTGTCAGTTACCAACGTCA
BMI1	CCATTGAATTCTTTGACCAGAA	AGCCACGTGCAGAAGGTC
cMYC	GGCTCCTGGCAAAAGGTCA	CTGCGTAGTTGTGCTGATGT
CYCLIN-D1	CCGTCCATGCGGAAGATC	GAAGACCTCCTCCTCGCACT
HES1	GAAGCACCTCCGGAACCT	GTCACCTCGTTCATGCACTC
ID1	GGTCCCTGATGTAGTCGATGA	CCAGAACCGCAAGGTGAG
MDK	ACCAAAGCAAAGGCCAAAG	ACTGGTGGGTCACATCTCG
MSI1	CCAATGGGTACCACTGAAGC	CACTCGTGGTCCTCAGTCAG
NESTIN	TGCGGGCTACTGAAAAGTTC	TGTAGGCCCTGTTTCTCCTG
NOTCH3	GCCAAGCGGCTAAAGGTA	CACTGACGGCAATCCACA
SOX1	AGACCTAGATGCCAACAATTGG	GCACCACTACGACTTAGTCCG
SOX2	GGGGGAATGGACCTTGTATAG	GCAAAGCTCCTACCGTACCA
SOX3	GACCTGTTCGAGAGAACTCATCA	CGGGAAGGGTAGGCTTATCAA
SOX4	AGCCGGAGGAGGAGATGT	TTCTCGGGTCATTTCCTAGC
SOX5	CTCGGCAAATGAAGGAGCAACTC	ACTGCCAGTTGCTGAGTCAGAC
SOX6	GGCGTCCCCCTACCCTGTCATCC	TGCTGCACACGGCTCCTCAC
SOX8	ACAACGCCGAGCTCAGCAAGAC	TCCTTCTTGTGCTGCACGCGAA
SOX9 (human)	GTACCCGCACTTGCACAAC	TCTCGCTCTCGTTCAGAAG
Sox9 (mouse)	GTACCCGCATCTGCACAAC	CTCCTCCACGAAGGGTCTCT
SOX11	AACTGGAGGTCCTGTTGTCG	GGAGGAGGAGGTGAGAAAGG
SOX12	TTCGAGTTCCCGGACTACTG	GGTGGGCTCAGTAGGTGAAA
SOX15	AAGTTCCTCGGCAACGACT	GAAGCGTCGATCCTGAAAAT
SOX17	ACGCTTTCATGGTGTGGGCTAAG	GTCAGCGCCTTCCACGACTTG
SURVIVIN	GCCCAGTGTTTCTTCTGCTT	AACCGGACGAATGCTTTTTA

Supplementary methods Table 1: Primers used for qPCR experiments.

Primary antibody	Host specie	Dilution	Provider	Reference
LC3B (I/II) (MAP1LC3B)	rabbit	1:350	Sigma	L7543
LC3B (I/II) (MAP1LC3B)	mouse	1:100	Sigma	M152-3
MSI1	mouse	1:100	Santa Cruz Biotechnology	sc-135721
NESTIN	rabbit	1:500	Abcam	ab105389
SOX9	rabbit	1:300	Millipore	AB5535

Supplementary methods Table 2: Antibodies used for immunofluorescence experiments.

Primary antibody	Host specie	Dilution	Provider	Reference
AKT	rabbit	1:1000	Cell Signaling	#9272
ALK	mouse	1:500	Santa Cruz Biotechnology	sc-398791
ATG5	rabbit	1:1000	Cell Signaling	#12994
ATG7	rabbit	1:1000	Santa Cruz Biotechnology	sc-33211
CD133	rabbit	1:500	Invitrogen	PA5-38014
Cleaved CASPASE-3 (D175)	rabbit	1:500	Cell Signaling	#9661
Cleaved PARP (D214)	rabbit	1:1000	Cell Signaling	#9541
ERK	mouse	1:1000	Cell Signaling	#4696
GAPDH	mouse	1:2000	Sigma	G8795
HSP90	mouse	1:1000	Santa Cruz Biotechnology	sc-69703
LC3B (I/II) (MAP1LC3B)	rabbit	1:3000	Sigma	L7543
MUSASHI-1 (MSI1)	mouse	1:750	Santa Cruz Biotechnology	sc-135721
NESTIN	rabbit	1:500	Abcam	ab105389
pAKT (S473)	rabbit	1:1000	Cell Signaling	#9271
pALK (Y1604)	rabbit	1:250	Cell Signaling	#3341S
pERK 1/2 (T202/Y204)	rabbit	1:1000	Cell Signaling	#9102
pS6 (S235/236)	rabbit	1:1000	Cell Signaling	#2211S
pSTAT3 (Y705)	rabbit	1:1000	Cell Signaling	#9145
pTSC2 (T1462)	rabbit	1:1000	Cell Signaling	#3617
S6	rabbit	1:1000	Cell Signaling	#2217
SOX2	mouse	1:1000	R&D Systems	MAB2018
SOX4	rabbit	1:500	Diagenode	CS129-100
SOX9	rabbit	1:1000	Millipore	AB5535
STAT3	rabbit	1:1000	Santa Cruz Biotechnology	sc-69703
TSC2	rabbit	1:1000	Cell Signaling	#3612
α-TUBULIN	mouse	1:5000	Sigma	T9026
β-ACTIN	mouse	1:5000	Sigma	A5441

Supplementary methods Table 3: Antibodies used for Western blot experiments.

Additional Supplementary information

LDA Statistics:

- Figure 3D:

Group 1	Group 2	Chisq	DF	Pr(>Chisq)
VEH CP	SOX9	0.498	1	0.48
VEH CP	MDK Ab CP	16.6	1	4.53e-05
VEH CP	MDK Ab SOX9	3.32	1	0.0683
SOX9	MDK Ab SOX9	1.28	1	0.257
MDK Ab CP	MDK AbSOX9	5.57	1	0.0183

- Figure 5D:

GH2

Group 1	Group 2	Chisq	DF	Pr(>Chisq)
VEH	Crizotinib	10.4	1	0.00126
VEH	TMZ	3.51	1	0.061
VEH	COMB	61.7	1	3.95E-15
Crizotinib	COMB	24.2	1	8.48E-07
TMZ	COMB	88.9	1	4.21E-21

12O12

Group 1	Group 2	Chisq	DF	Pr(>Chisq)
VEH	Crizotinib	28.9	1	7.64E-08
VEH	TMZ	0.139	1	0.71
VEH	COMB	54.3	1	1.68E-13
Crizotinib	COMB	5.47	1	0.0193
TMZ	COMB	50.1	1	1.44E-12

- Figure 5E:

Group 1	Group 2	Chisq	DF	Pr(>Chisq)
VEH	THC:CBD	7.72	1	0.00546
VEH	MDK Ab.	20.8	1	5.18e-06
VEH	COMB	71	1	3.59e-17
MDK Ab.	COMB	20.4	1	6.39e-06
THC:CBD	COMB	36.8	1	1.33e-09

Group 1	Group 2	Chisq	DF	Pr(>Chisq)
VEH	THC:CBD	7.72	1	0.00546
VEH	Crizotinib	26.9	1	2.14e-07
VEH	COMB	71	1	3.59e-17
Crizotinib	COMB	16.0	1	6.27e-05
THC:CBD	COMB	32.3	1	1.48e-08

- Figure 4D:

Group 1	Group 2	Chisq	DF	Pr(>Chisq)
VEH	VEH shATG5	0.58	1	0.446
VEH	MDK Ab.	7.35	1	0.00672
VEH	MDK Ab shATG5	0.0198	1	0.888
shATG5	MDK Ab shATG5	0.407	1	0.524
MKab	MDK Ab shATG5	6.93	1	0.00847

- Supplementary Figure 5G:

Group 1	Group 2	Chisq	DF	Pr(>Chisq)
Control	SOX9	0.029	1	0.865
Control	shMDK	5.32	1	0.0211
Control	shMDK SOX9	0.106	1	0.745
SOX9	shMDK SOX9	0.0242	1	0.876
shMDK	shMDK SOX9	4.1	1	0.043

- Supplementary Figure 7C:

Group 1	Group 2	Chisq	DF	Pr(>Chisq)
VEH	VEH shATG5	0.58	1	0.446
VEH	CRIZO	21.9	1	2.91e-06
VEH	CRIZO shATG5	0.155	1	0.694
sh ATG5	CRIZO shATG5	0.131	1	0.717
CRIZO	CRIZO shATG5	18.2	1	1.96e-05

- Supplementary Figure 7D:

Group 1	Group 2	Chisq	DF	Pr(>Chisq)
VEH shC	VEH shATG7	5.82	1	0.0158
VEH shC	MDKab shC	26.2	1	3.14e-07
VEHshC	MDKab shATG7	4.71	1	0.0300
VEH shATG7	MDKab shATG7	0.056	1	0.813
MDKab shC	MDKab shATG7	10.2	1	0.00139

Group 1	Group 2	Chisq	DF	Pr(>Chisq)
VEH shC	VEH shATG7	5.82	1	0.0158
VEH shC	CRIZO shC	43.9	1	3.47e-11
VEH shC	CRIZO shATG7	3.92	1	0.0479
VEH shATG7	CRIZO shATG7	0.207	1	0.649
CRIZO shC	CRIZO shATG7	25.6	1	4.23e-07

- Supplementary Figure 9C:

Group 1	Group 2	Chisq	DF	Pr(>Chisq)
VEH	THC	0.771	1	0.38
VEH	MDK Ab.	39.9	1	2.67E-10
VEH	COMB	64.4	1	1.01E-15
MDK Ab.	COMB	4.58	1	0.0324
THC	COMB	49.5	1	2.03E-12

Group 1	Group 2	Chisq	DF	Pr(>Chisq)
VEH	THC	1.77	1	0.183
VEH	Crizotinib	24.7	1	6.82E-07
VEH	COMB	77.6	1	1.24E-18
Crizotinib	COMB	18.9	1	1.40E-05
THC	COMB	58.3	1	2.25E-14

Full uncut gels

Fig. 2A:

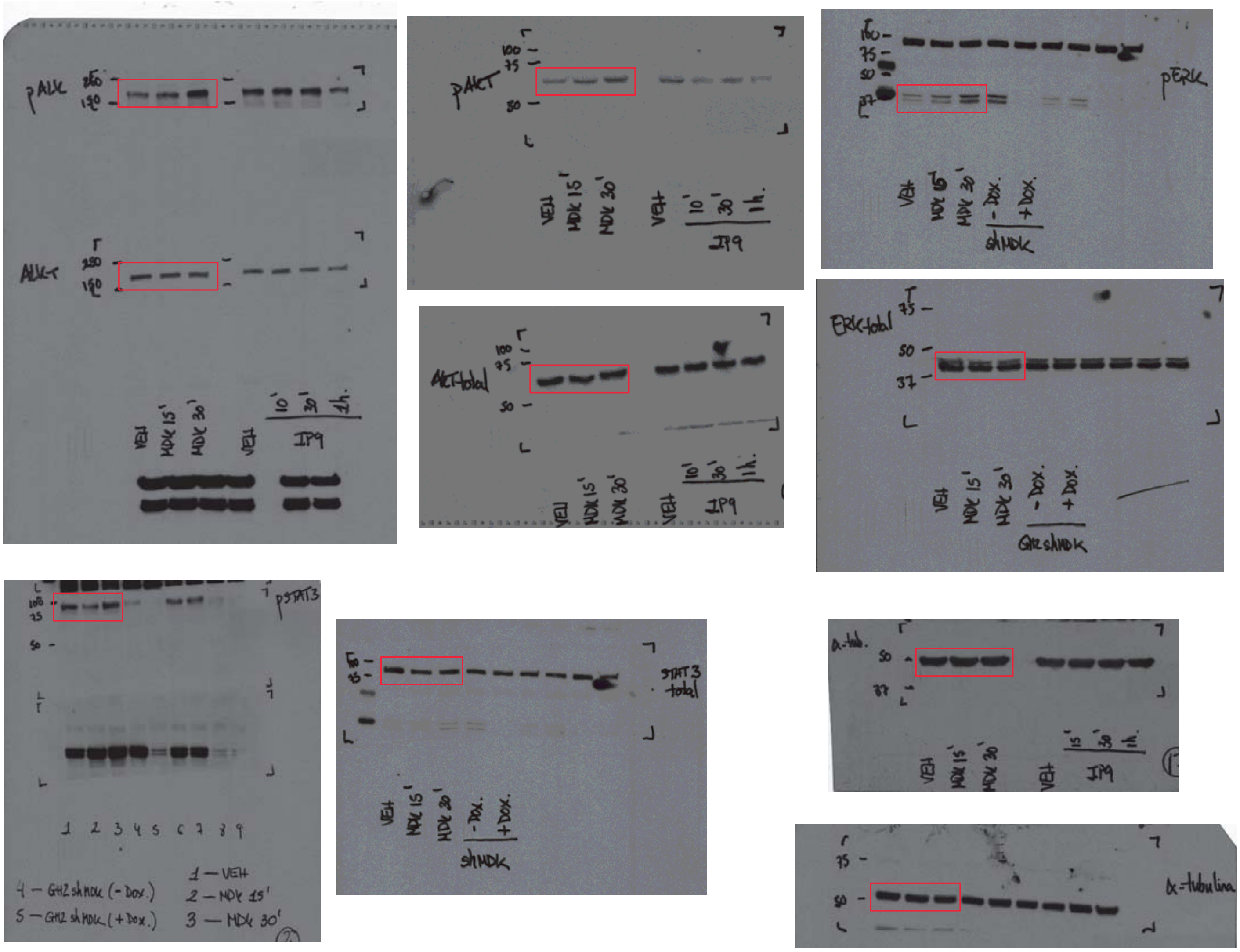
pALK
ALK-t
pAKT
AKT-total
pERK
ERK-total
pSTAT3
STAT3 total
α-tubulina
VEH
MDK 15'
MDK 30'
IP9
shMDK
- Dox.
+ Dox.
1 — VEH
2 — MDK 15'
3 — MDK 30'
4 — GH2 shMDK (- Dox.)
5 — GH2 shMDK (+ Dox.)

Fig. 2B:

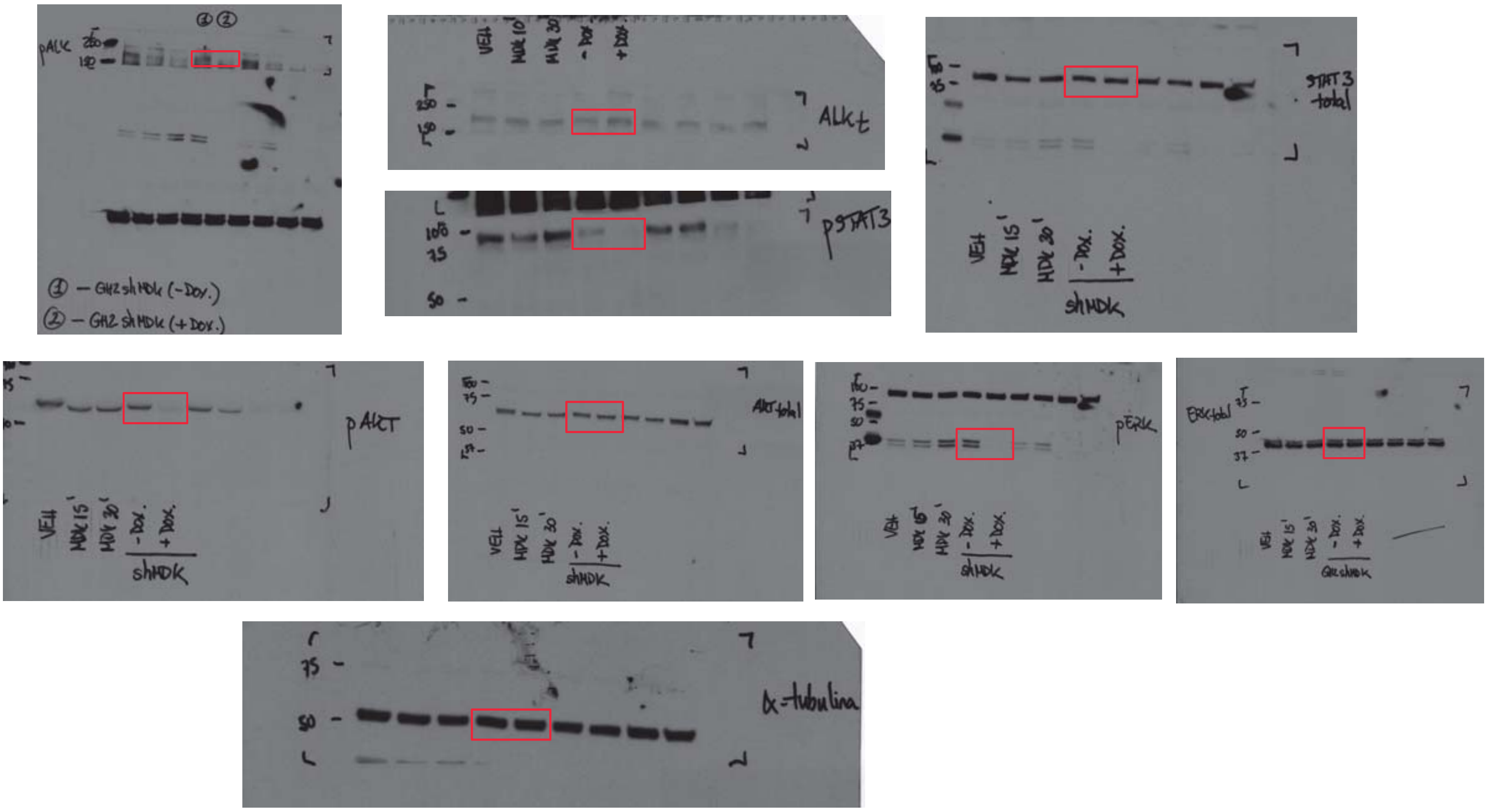
pALK
ALKt
pSTAT3
STAT3 total
pAKT
AKT-total
pERK
ERK-total
α-tubulina
① — GH2 shMDK (- Dox.)
② — GH2 shMDK (+ Dox.)
VEH
MDK 15'
MDK 30'
shMDK
- Dox.
+ Dox.

Fig. 3A:

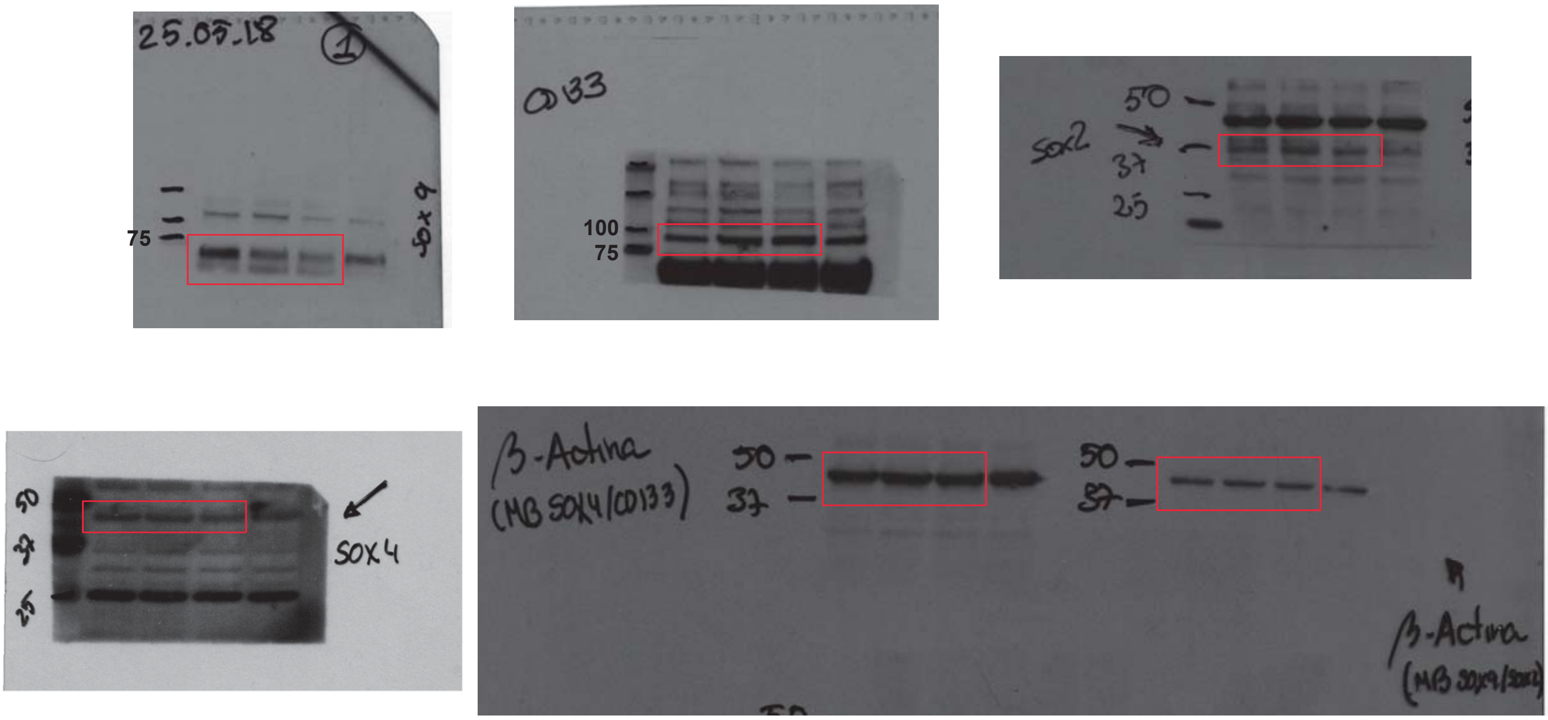

Fig. 4C:

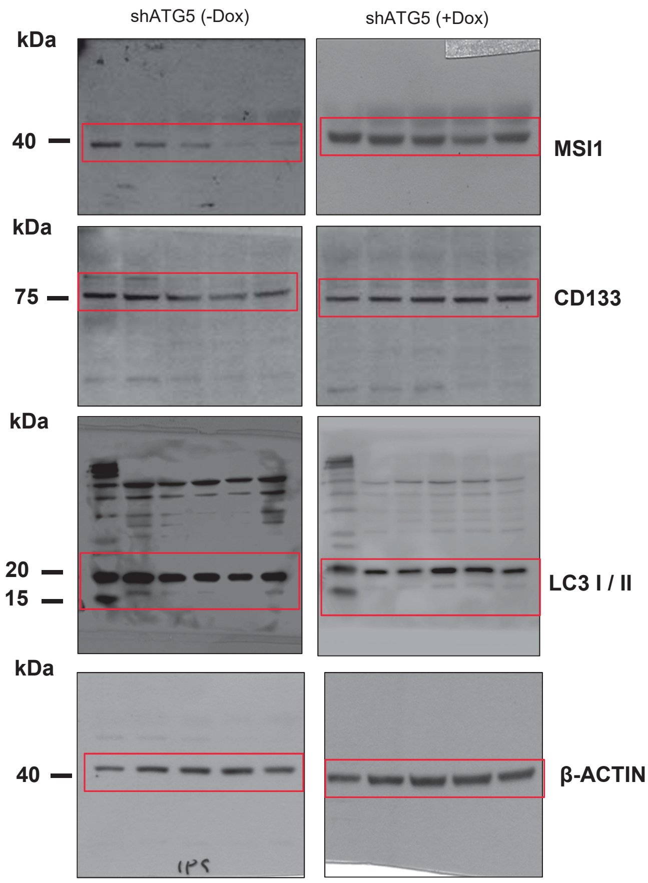

Fig. 4E:

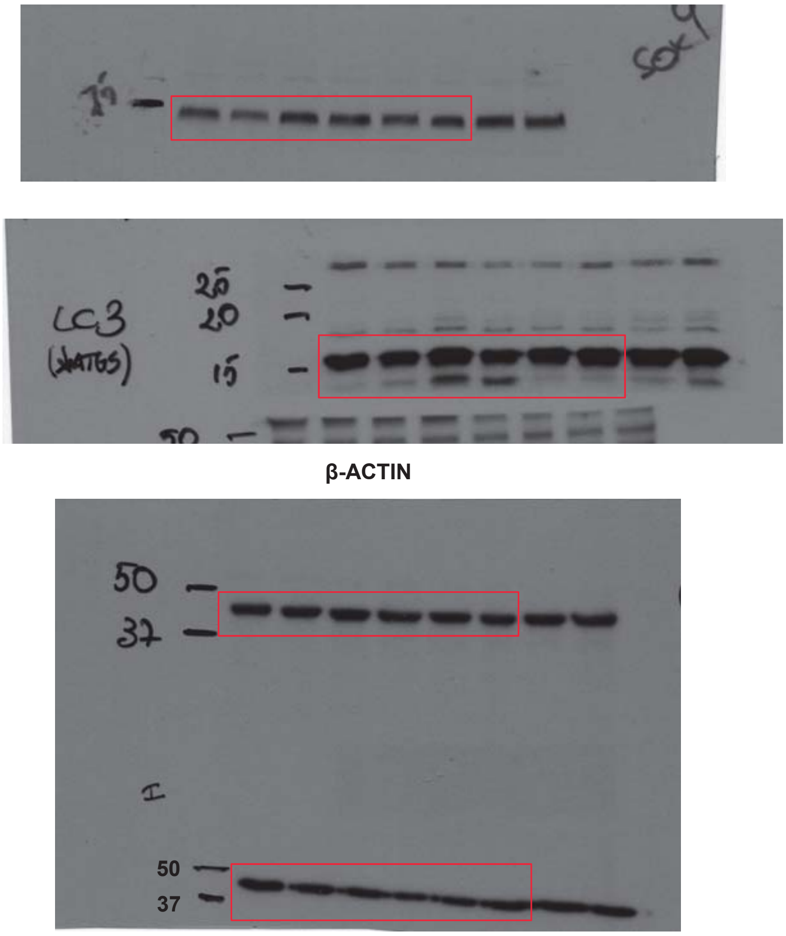

Fig. 4F:

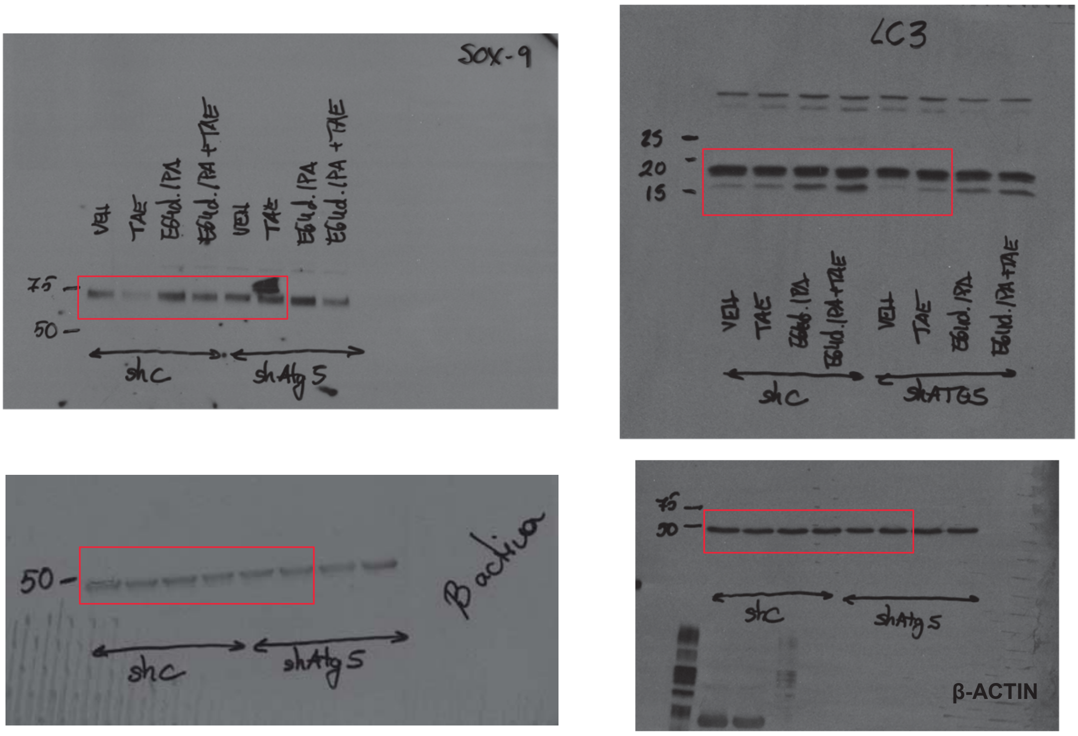
SOX-9
VEH
TAE
shC
shAtg5
LC3
β actina
β-ACTIN

Fig. 5C:

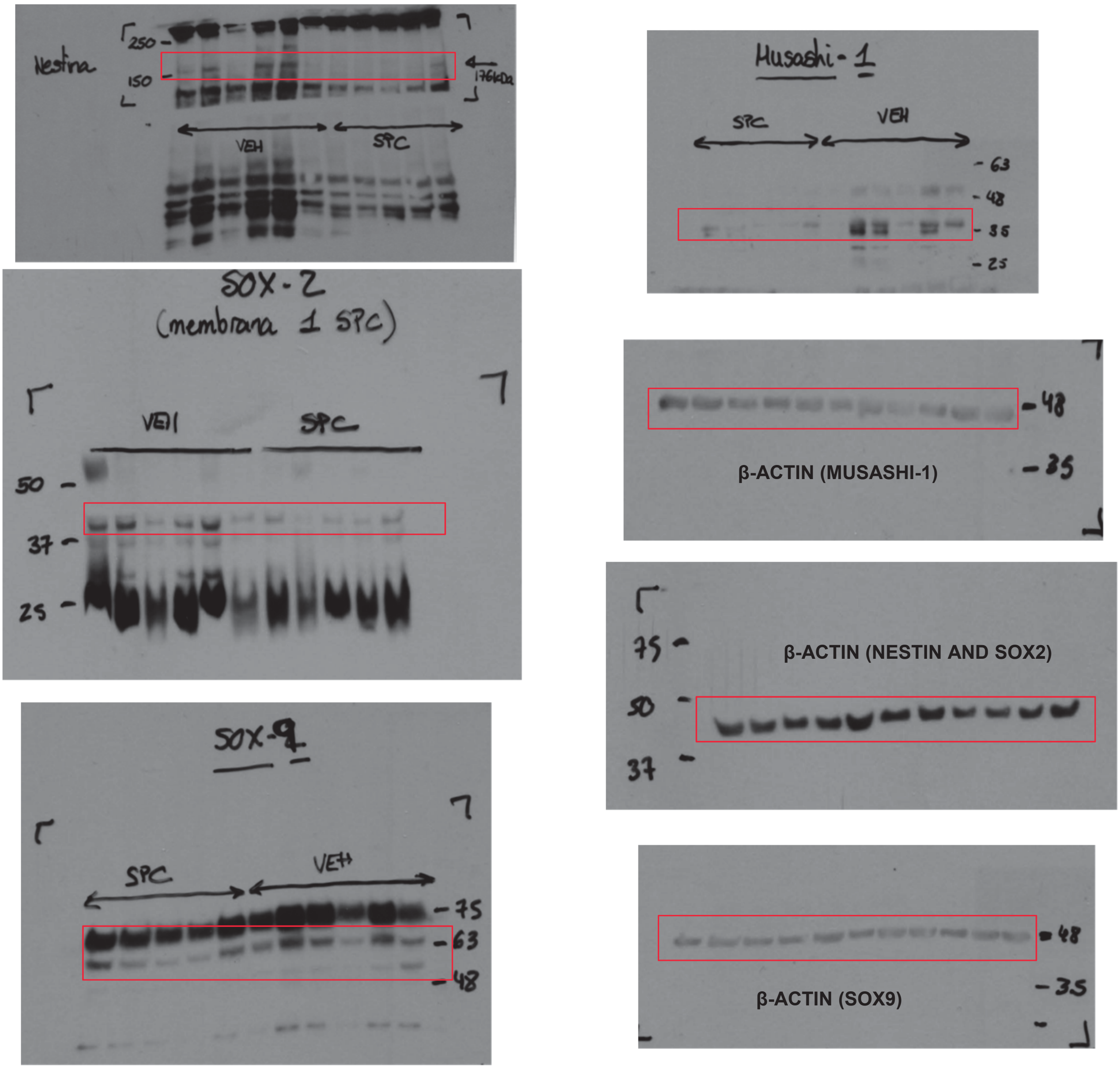
Nestina
176kDa
VEH
SPC
Musashi-1
SOX-2
(membrana 1 SPC)
SOX-9
β-ACTIN (MUSASHI-1)
β-ACTIN (NESTIN AND SOX2)
β-ACTIN (SOX9)

Fig. S2A:

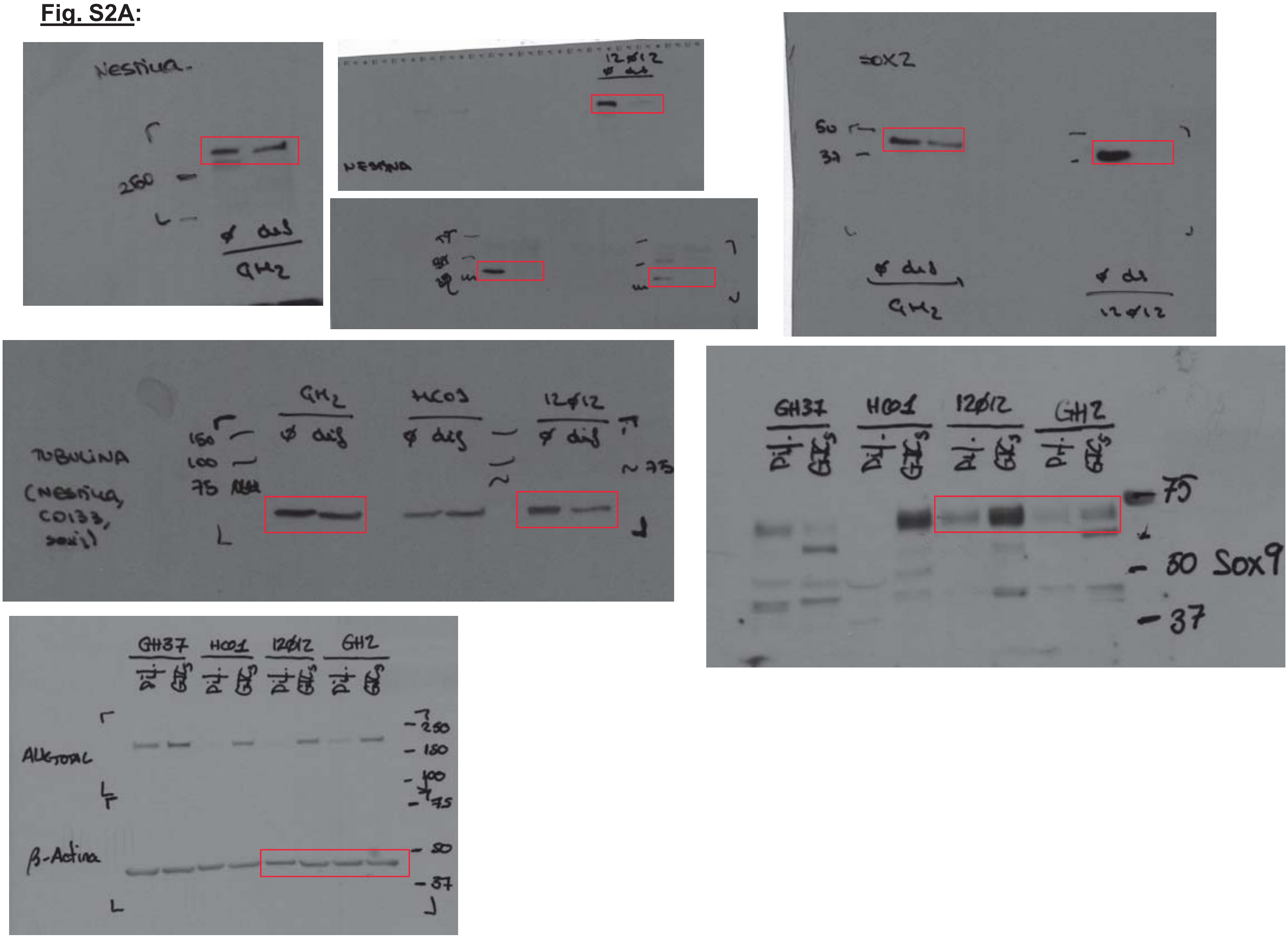

Fig. S3F:

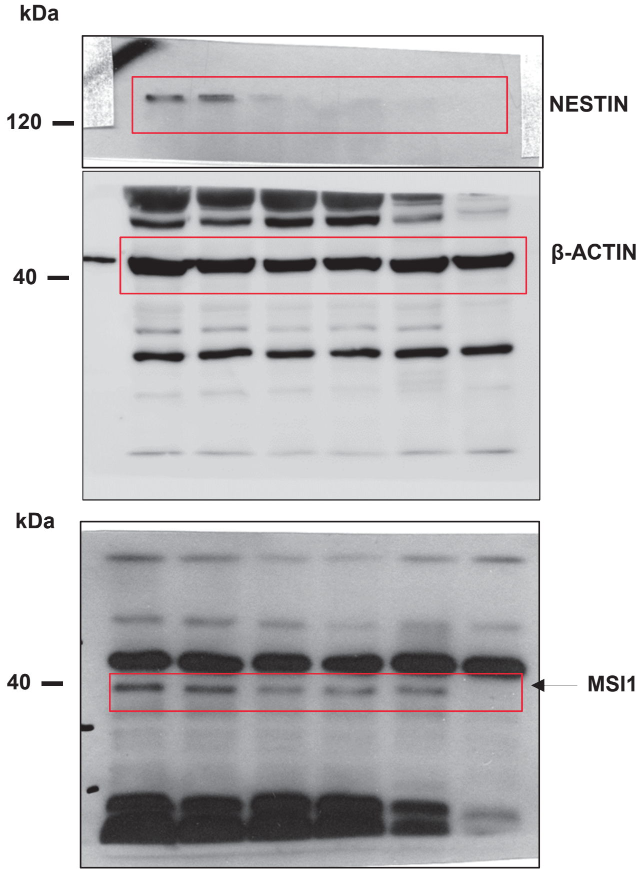

Fig. S4A:

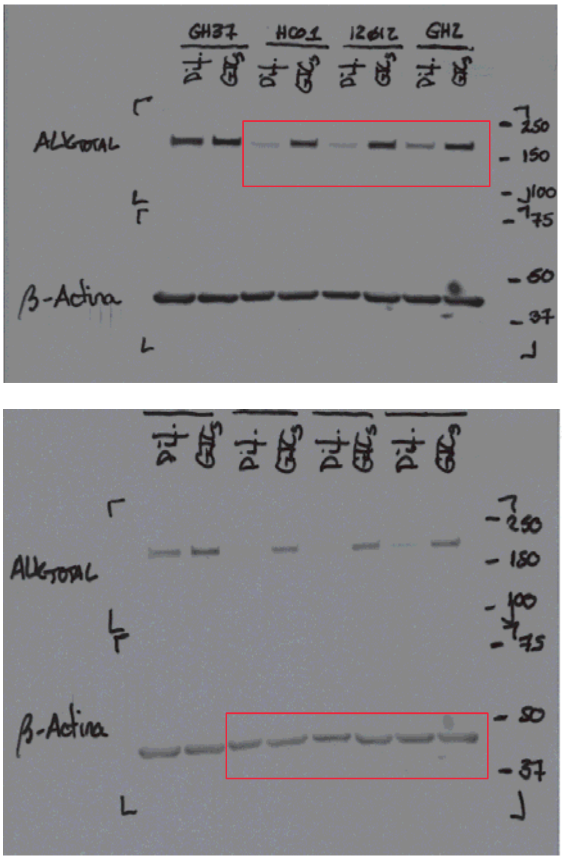

Fig. S4B:

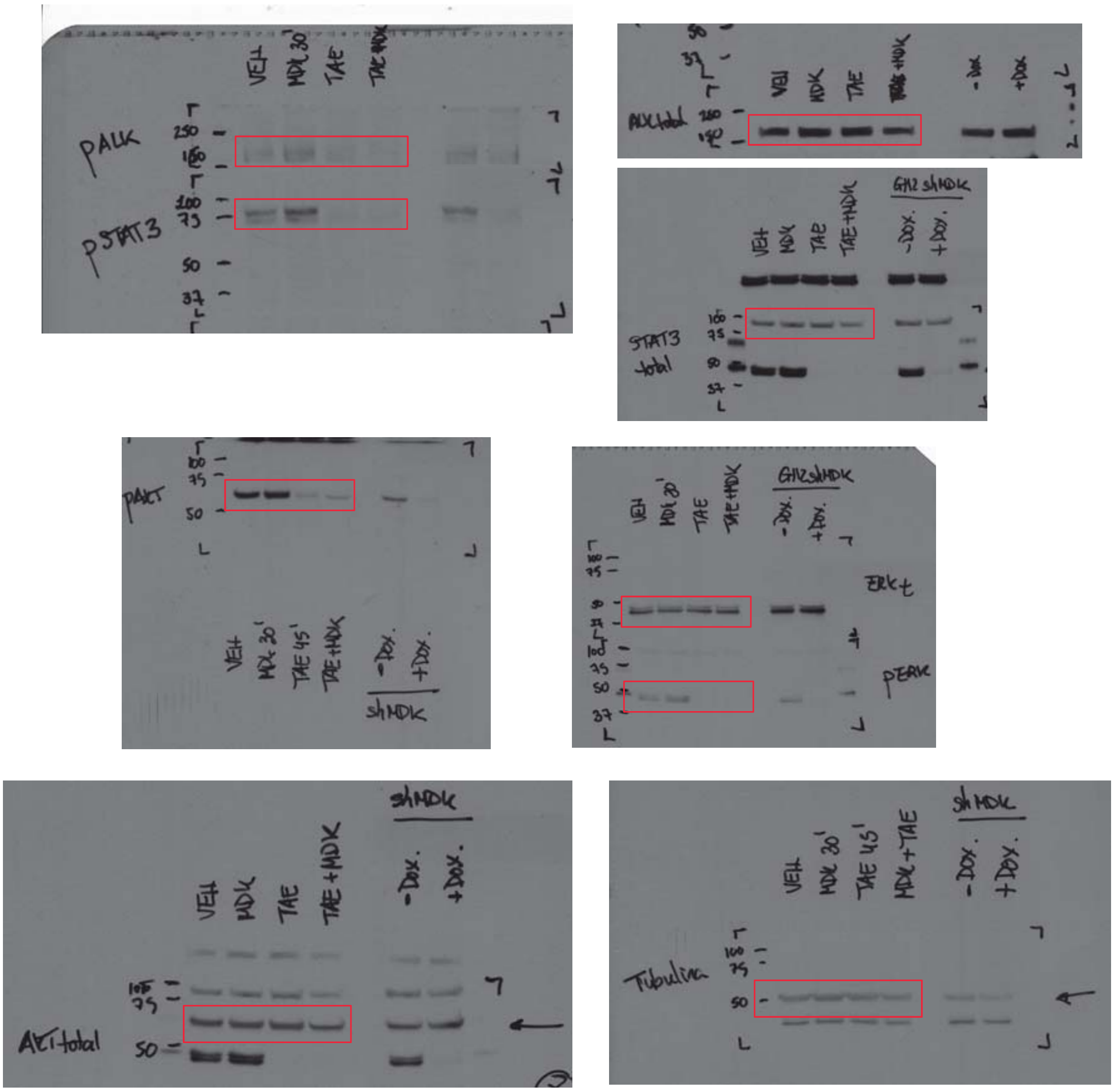

Fig. S5C:

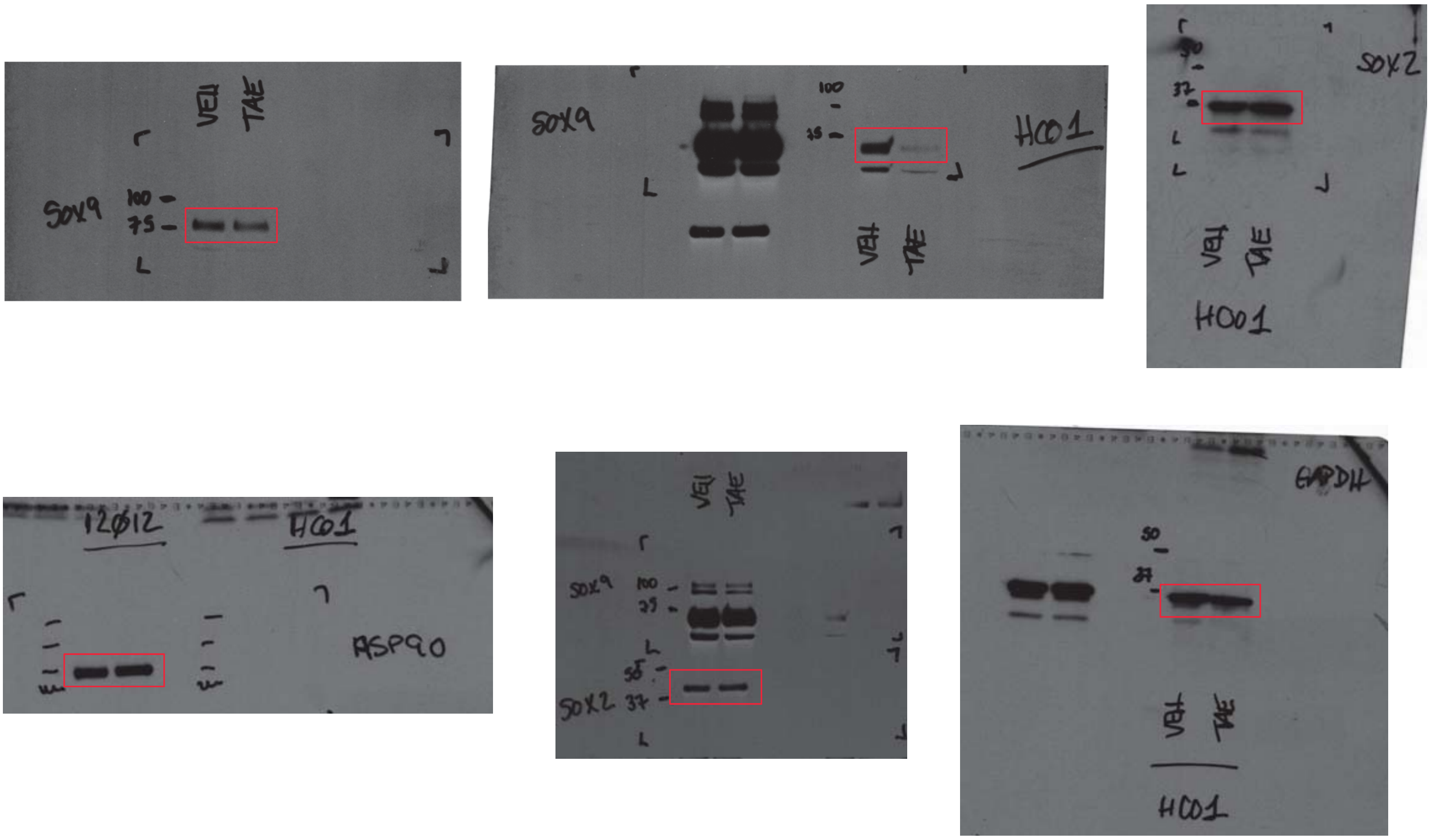

Fig. S6B:

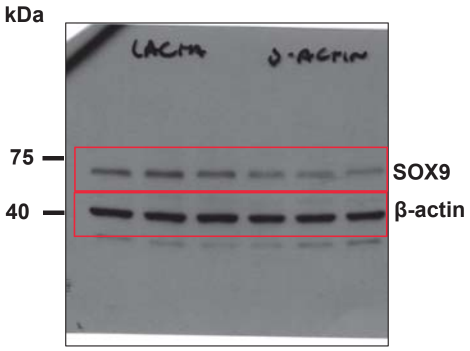

Fig. S6D:

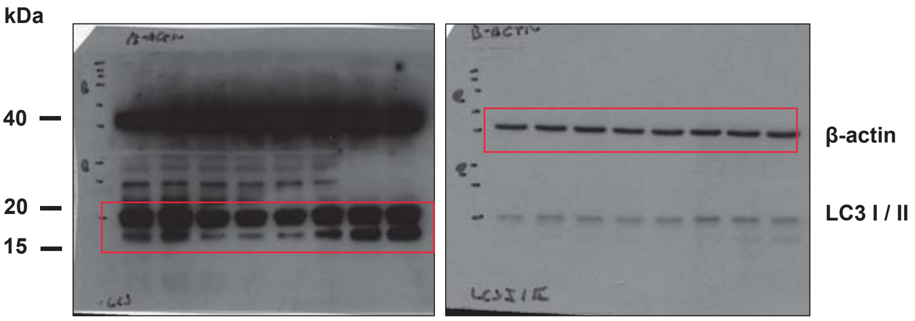

Fig. S6C:

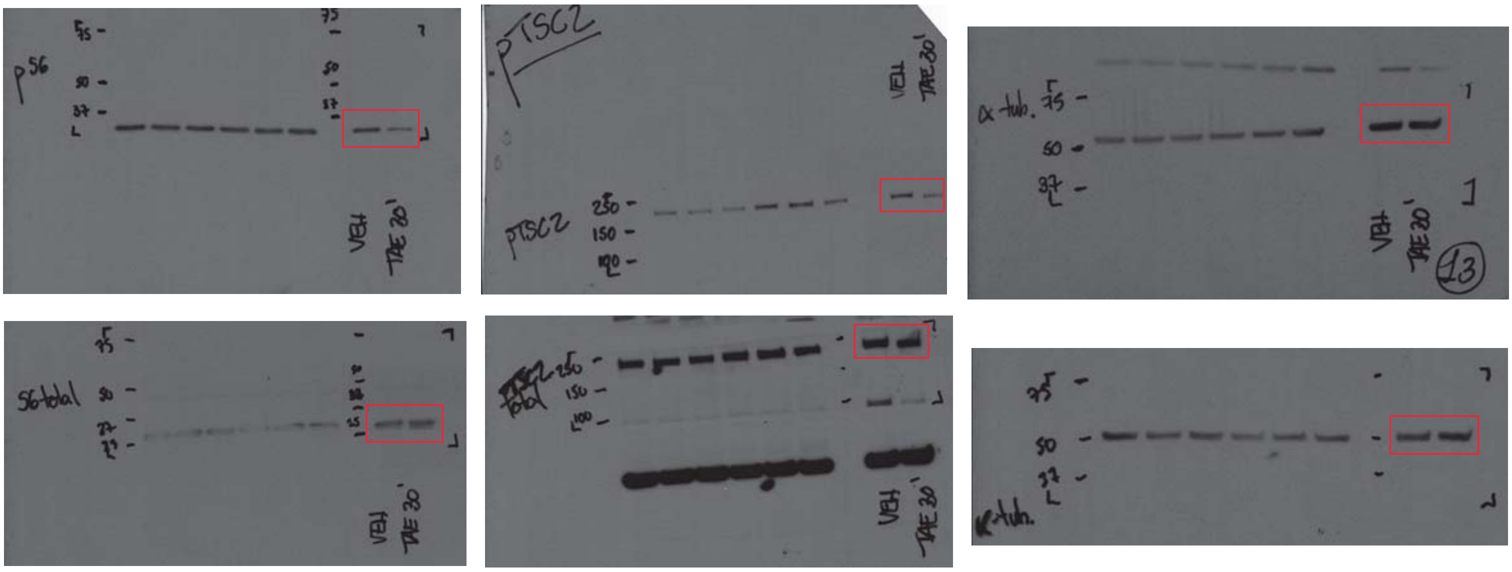

Fig. S7B:

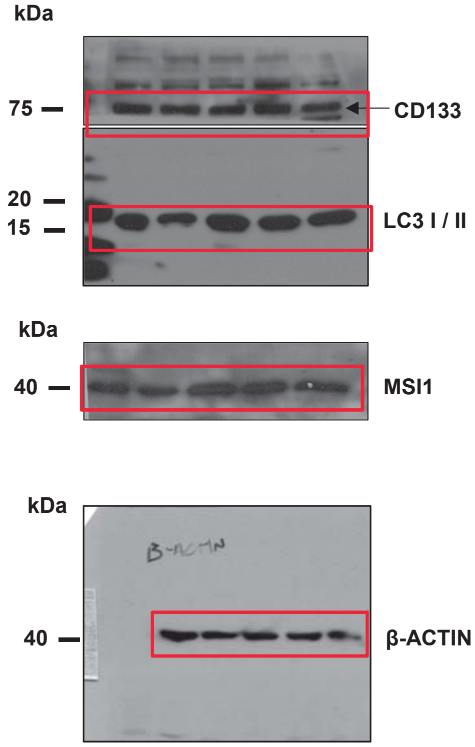

Fig. S7E:

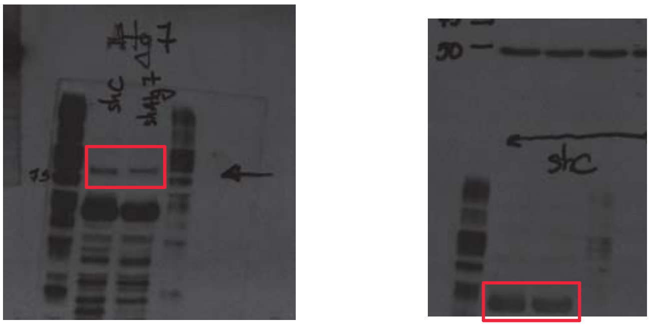

Fig. S7F:

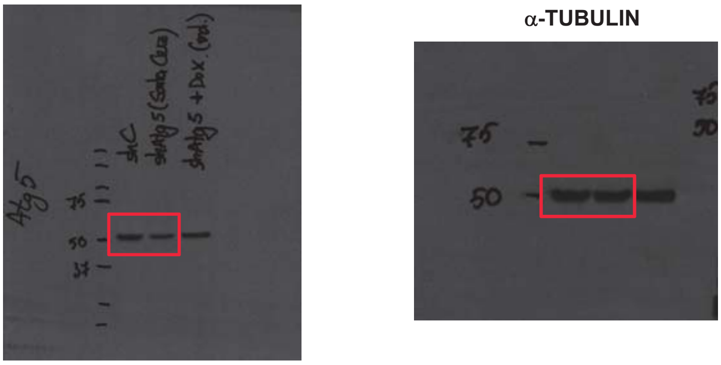

Fig. S7G:

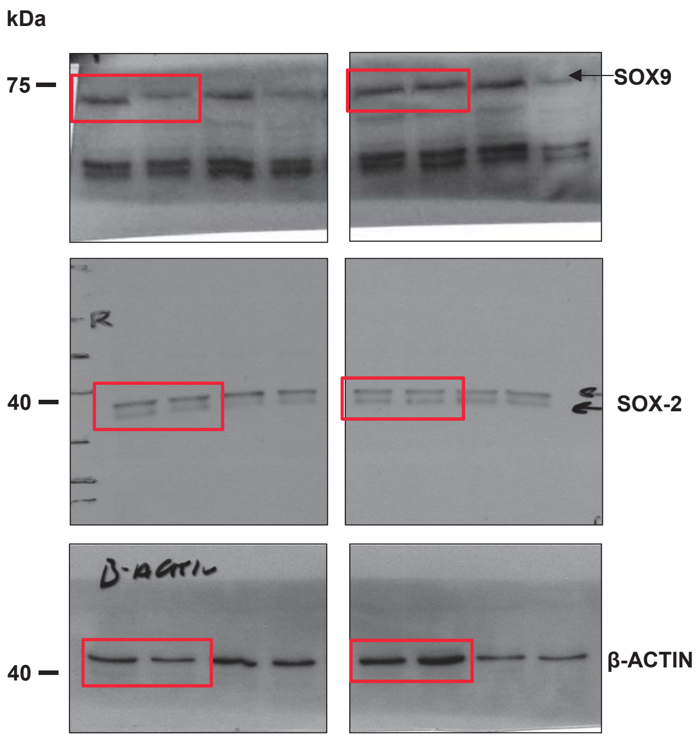

Fig. S7H:

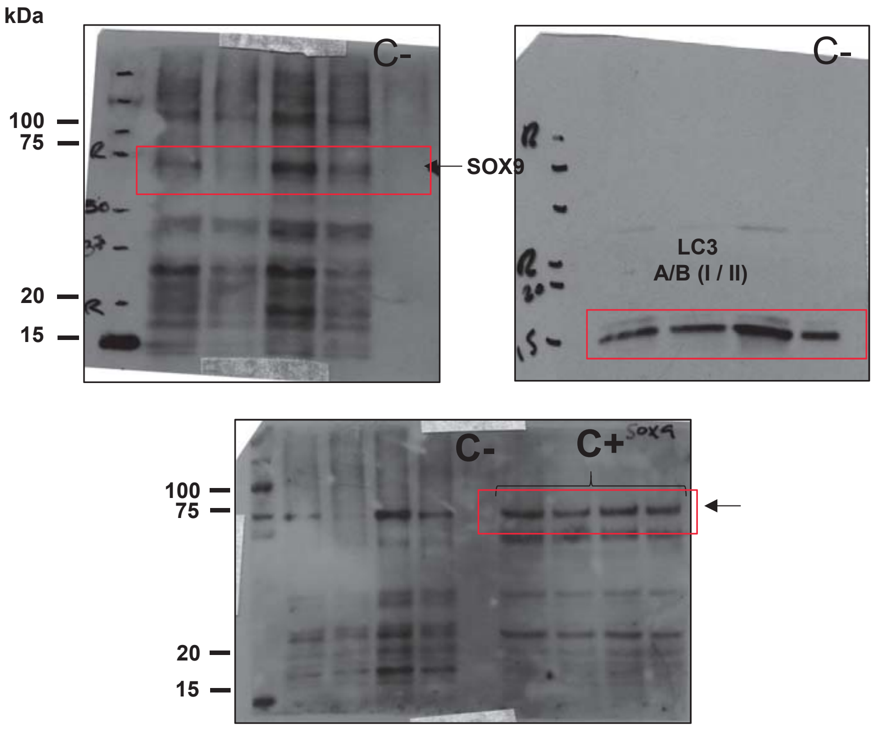

Fig. S7I:

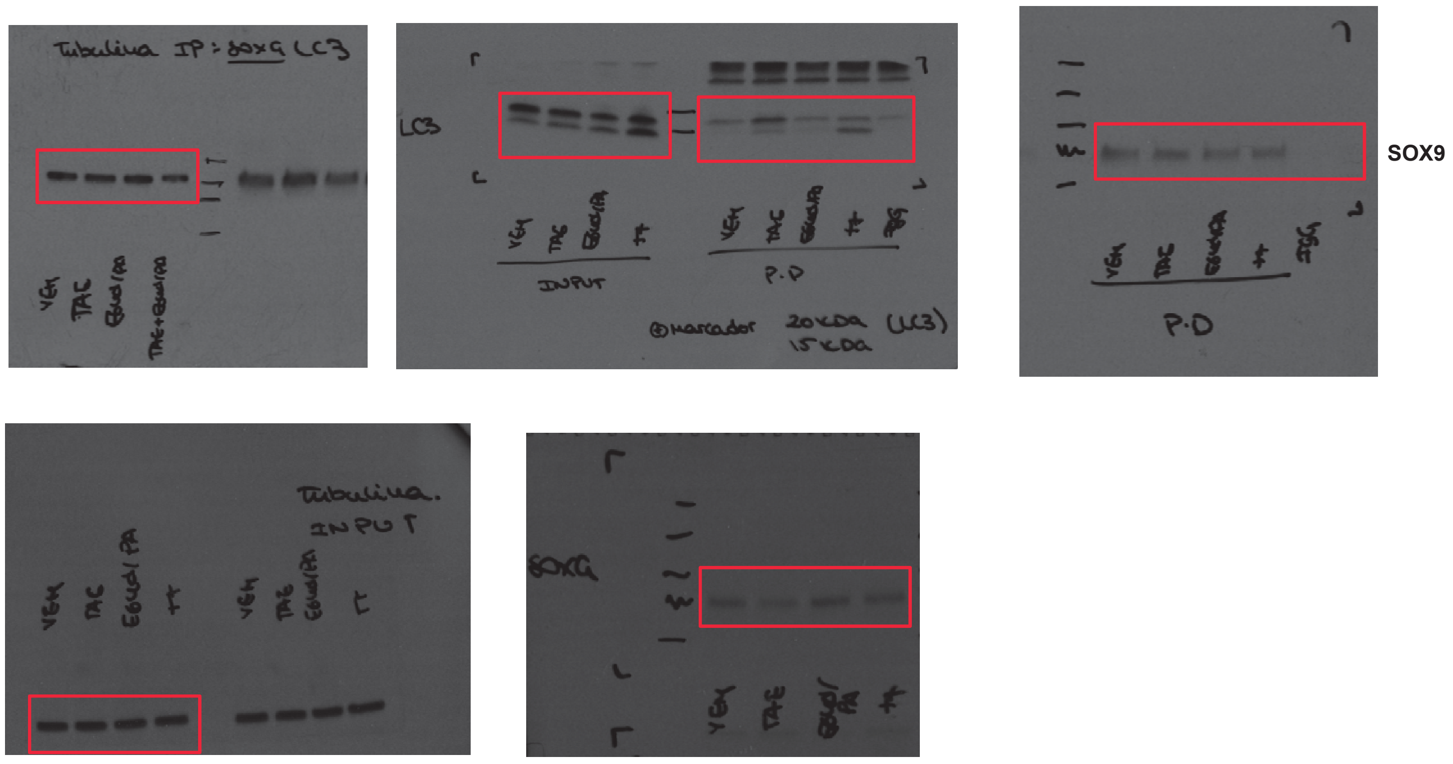

Fig. S9A:

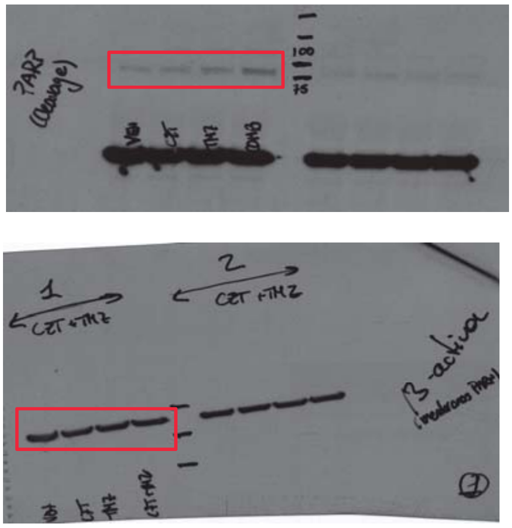

Fig. S9D:

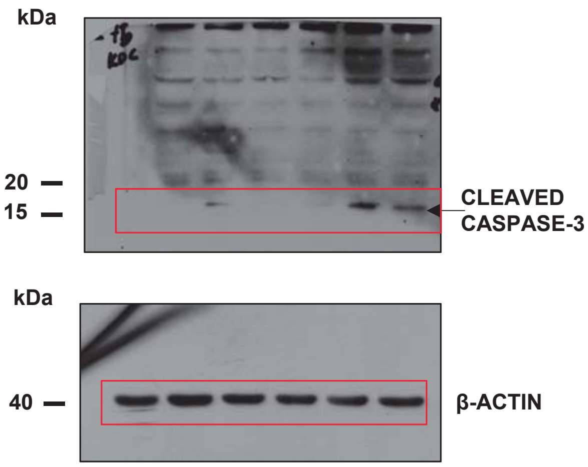

Supplement: Supplementary file 1 — Supplementary statistical analysis, methods, figures and tables. [file thnov10p5120s1.pdf]
